# The molecular classification of cancer‐associated fibroblasts on a pan‐cancer single‐cell transcriptional atlas

**DOI:** 10.1002/ctm2.1516

**Published:** 2023-12-26

**Authors:** Bonan Chen, Wai Nok Chan, Fuda Xie, Chun Wai Mui, Xiaoli Liu, Alvin H. K. Cheung, Raymond W. M. Lung, Chit Chow, Zhenhua Zhang, Canbin Fang, Peiyao Yu, Shihua Shi, Shikun Zhou, Guoming Chen, Zhangding Wang, Shouyu Wang, Xiaofan Ding, Bing Huang, Li Liang, Yujuan Dong, Chi Chun Wong, William K. K. Wu, Alfred S. L. Cheng, Nathalie Wong, Jun Yu, Kwok Wai Lo, Gary M. K. Tse, Wei Kang, Ka Fai To

**Affiliations:** ^1^ Department of Anatomical and Cellular Pathology State Key Laboratory of Translational Oncology Sir Y.K. Pao Cancer Center The Chinese University of Hong Kong Hong Kong China; ^2^ Institute of Digestive Disease State Key Laboratory of Digestive Disease Li Ka Shing Institute of Health Science The Chinese University of Hong Kong Hong Kong China; ^3^ CUHK‐Shenzhen Research Institute Shenzhen China; ^4^ School of Pharmaceutical Sciences Sun Yat‐Sen University Guangzhou China; ^5^ Department of Pathology Guangdong Province Key Laboratory of Molecular Tumor Pathology Nanfang Hospital and Basic Medical College Southern Medical University Guangzhou China; ^6^ Hospital of Chengdu University of Traditional Chinese Medicine Chengdu China; ^7^ Friedrich Miescher Institute for Biomedical Research Basel Switzerland; ^8^ School of Biomedical Sciences and Engineering South China University of Technology Guangzhou China; ^9^ School of Chinese Medicine Li Ka Shing Faculty of Medicine The University of Hong Kong Hong Kong China; ^10^ Department of Gastroenterology The Affiliated Drum Tower Hospital of Nanjing University Medical School Nanjing China; ^11^ Department of Hepatobiliary Surgery The Affiliated Drum Tower Hospital of Nanjing University Medical School Nanjing China; ^12^ Faculty of Health Sciences University of Macau Macao China; ^13^ Guangdong Provincial Key Laboratory of Gastroenterology Department of Gastroenterology Nanfang Hospital Southern Medical University Guangzhou China; ^14^ Department of Surgery The Chinese University of Hong Kong Hong Kong China; ^15^ Department of Anaesthesia and Intensive Care The Chinese University of Hong Kong Hong Kong China; ^16^ School of Biomedical Sciences The Chinese University of Hong Kong Hong Kong China; ^17^ Department of Medicine and Therapeutics The Chinese University of Hong Kong Hong Kong China

**Keywords:** cancer‐associated fibroblast, molecular classification, pan‐cancer, single‐cell transcriptional atlas

## Abstract

**Background:**

Cancer‐associated fibroblasts (CAFs), integral to the tumour microenvironment, are pivotal in cancer progression, exhibiting either pro‐tumourigenic or anti‐tumourigenic functions. Their inherent phenotypic and functional diversity allows for the subdivision of CAFs into various subpopulations. While several classification systems have been suggested for different cancer types, a unified molecular classification of CAFs on a single‐cell pan‐cancer scale has yet to be established.

**Methods:**

We employed a comprehensive single‐cell transcriptomic atlas encompassing 12 solid tumour types. Our objective was to establish a novel molecular classification and to elucidate the evolutionary trajectories of CAFs. We investigated the functional profiles of each CAF subtype using Single‐Cell Regulatory Network Inference and Clustering and single‐cell gene set enrichment analysis. The clinical relevance of these subtypes was assessed through survival curve analysis. Concurrently, we employed multiplex immunofluorescence staining on tumour tissues to determine the dynamic changes of CAF subtypes across different tumour stages. Additionally, we identified the small molecule procyanidin C1 (PCC1) as a target for matrix‐producing CAF (matCAF) using molecular docking techniques and further validated these findings through in vitro and in vivo experiments.

**Results:**

In our investigation of solid tumours, we identified four molecular clusters of CAFs: progenitor CAF (proCAF), inflammatory CAF (iCAF), myofibroblastic CAF (myCAF) and matCAF, each characterised by distinct molecular traits. This classification was consistently applicable across all nine studied solid tumour types. These CAF subtypes displayed unique evolutionary pathways, functional roles and clinical relevance in various solid tumours. Notably, the matCAF subtype was associated with poorer prognoses in several cancer types. The targeting of matCAF using the identified small molecule, PCC1, demonstrated promising antitumour activity.

**Conclusions:**

Collectively, the various subtypes of CAFs, particularly matCAF, are crucial in the initiation and progression of cancer. Focusing therapeutic strategies on targeting matCAF in solid tumours holds significant potential for cancer treatment.

## Graphical abstract

1. At the single‐cell level, we identified four CAF subtypes in 12 solid tumours: proCAF, iCAF, myCAF and matCAF.

2. Each subtype exhibited different evolutionary trajectories during cancer progression.

3. iCAF was markedly absent in ‘cold’ tumour types, which typically respond poorly to immunotherapy.

4. Signatures associated with matCAF were consistently correlated with unfavourable prognoses across various cancers.

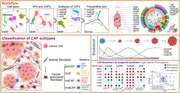

## 1 INTRODUCTION

Solid tumours consist of both parenchymal tumour cells and a diverse array of stromal cells within the tumour microenvironment (TME).^1^ Cancer‐associated fibroblasts (CAFs) stand out in the TME as pivotal influences on cancer progression, performing functions that can either support or inhibit tumour growth.^2^, ^3^ Being the main sources for the extracellular matrix (ECM), along with growth factors, cytokines, chemokines as well as other stimulating molecules, CAFs play a pivotal role in shaping cancer evolution, vasculogenesis, metastasis, immune modulation, metabolic reprogramming and resistance to treatment.^4^, ^5^, ^6^

Despite the existence of well‐recognised CAF markers such as α‐smooth muscle actin (α‐SMA), fibroblast activation protein (FAP), and fibroblast‐specific protein‐1 (S100A4) that facilitate CAF identification, the intrinsic heterogeneity of CAFs presents a challenge in specifying distinct CAF populations.^7^, ^8^, ^9^ Recent research suggests that a more refined combination of markers might be necessary to accurately identify these populations.^10^ In various cancers, specific subtypes of CAFs have been identified, each with unique roles in cancer progression or immune suppression.^11^, ^12^, ^13^, ^19^ However, the broad heterogeneity of CAFs complicates the development of a consistent classification system across different human cancers. Additionally, the dynamic nature of tumour tissues, particularly in terms of drug resistance or metastatic tendencies, underscores the critical role of CAFs in tumour adaptation.^4^, ^11^, ^13^, ^14^ Despite the varied landscape of CAFs, these cells often originate from a similar source, and their evolutionary trajectory is closely linked with tumour progression.^15^, ^16^

In this study, we utilised a pan‐cancer single‐cell transcriptional atlas to introduce a new molecular categorisation for CAFs, shedding light on their evolution and clinical significance. By employing single‐cell RNA sequencing (scRNA‐seq) datasets from nine varieties of solid tumours, we discovered four distinct molecular CAF clusters: progenitor CAF (proCAF), inflammatory CAF (iCAF), myofibroblastic CAF (myCAF) and matrix‐producing CAF (matCAF). Each subtype displays unique molecular characteristics, evolutionary trajectories, functional roles and clinical implications. Overall, our findings reveal the evolutionary path of CAF subpopulations in tumourigenesis and progression, offering new insights into the therapeutic targeting of CAFs in solid tumours.

## 2 MATERIALS AND METHODS

### 2.1 Cell lines, CAFs, primary samples and clinical cohorts

Cell isolation and culture in our study were executed in accordance with an established protocol.^18^ We began by enzymatically digesting clinical tumour samples using a combination of collagenase and hyaluronidase, facilitating cell dissociation. Following this, differential centrifugation was employed to separate stromal and epithelial components. The stromal fraction, which is rich in fibroblasts, was then cultured in a medium specifically designed for fibroblasts to enhance CAF populations. For the specific isolation and identification of matCAFs, we concentrated on the marker COL10A1, which is a distinctive feature of this subtype. After cultivation, we applied quantitative real‐time polymerase chain reaction and Western blot techniques to confirm the expression of COL10A1. matCAF cells with stable knockdown of COL10A1 were cultured using shRNA targeting COL10A1 (shCOL10A1, #sc‐9532‐SH, Santa Cruz Biotechnology).

Moreover, primary tumour tissues for LUAD, gastric cancer (STAD), breast cancer (BRCA) and colorectal cancer (COAD) were obtained from Prince of Wales Hospital. The study cohorts comprised formalin‐fixed paraffin‐embedded tissues from cancer patients diagnosed at Prince of Wales Hospital between 1999 and 2006 (Hong Kong cohort). The research received approval from the Joint Chinese University of Hong Kong‐New Territories East Cluster Clinical Research Ethics Committee (CREC Ref. No. 2022.060). Additionally, the scRNA‐seq samples for STAD research were sourced from the Affiliated Drum Tower Hospital of Nanjing University Medical School, with authorisation from its Ethics Committee.^19^ All procedures adhered strictly to ethical guidelines, safeguarding participant confidentiality and rights, and were in line with the principles of the Declaration of Helsinki.

### 2.2 scRNA‐seq dataset preprocessing, dimension reduction and clustering

For our scRNA‐seq analysis, the primary tool used was the ‘Seurat’ R package (version 4.0.2).^20^ The initial step in our analysis involved a detailed data preprocessing phase, which included several critical filtering actions: (a) eliminating cells that had detected less than 300 distinct genes; (b) discarding cells with mitochondrial transcript levels exceeding 10%; (c) filtering out cells where gene expression from red blood cell markers exceeded 3% of the total; and (d) removing genes that were expressed within three cells. The rigorous preprocessing was followed by log‐normalisation, scaling, and subsequent log‐transformation of the gene expression data. We then used the ‘FindVariableFeatures’ function in Seurat to identify the top 3000 genes with high variability in the dataset, selecting these using the ‘vst’ method. This step concluded by normalising and adjusting the scale of the expression data. To address the issue of intra‐heterogeneity, our methodology included comparing and integrating different patient samples within the same cancer type. The ‘FindIntegrationAnchors’ and ‘IntegrateData’ functions from Seurat were pivotal in this process. These functions identify common anchors between different samples, assign a relevance score to each anchor, and then execute a weighted integration, effectively combining the data to resemble a single sample. This approach is in line with contemporary approaches to data integration and analysis.^21^ Exact methods used include principal component analysis (PCA) and *k*‐nearest neighbour (KNN) clustering.

Post preprocessing, we conducted dimension reduction using PCA with the ‘RunPCA’ function. To ascertain the optimal number of principal components (PCs) for subsequent analysis, we referred to the ‘elbow’ point in the scree plot, created by the ‘ElbowPlot’ function. Within this reduced PCA space, clusters were discerned using the KNN algorithm, facilitated by ‘FindNeighbours’ and ‘FindClusters’ functions. We then visualised these clusters employing uniform manifold approximation and projection (UMAP) with the ‘RunUMAP’ and ‘DimPlot’ functions.

Following the identification of clusters, we determined the differentially expressed genes (DEGs) for each cluster. This was done through Wilcoxon rank sum test, executed by the ‘FindAllMarkers’ function. We selected DEGs based on a *p*‐value of less than .05 and visualised them using UMAP plots (‘FeaturePlot’), heatmaps (‘DoHeatmap’) and violin plots (‘VlnPlot’). We then grouped cell clusters based on gene expression similarities and their distribution patterns. In our single‐cell dataset analysis workflow, we first differentiated between various cell types. Subsequently, we distinguished normal fibroblasts (NFs) and CAFs within the isolated fibroblast cell population. The final step involved defining the subtypes of CAFs. The preprocessed and categorised scRNA dataset was then ready for further downstream analyses, which included trajectory analysis and exploration of cell–cell communication mechanisms.

### 2.3 Single‐cell trajectory analysis

The pseudotime analysis of single cells in our study was conducted using Monocle2,^22^ available in the ‘monocle’ R package (version 2.18.0). The process began with the creation of a monocle object using the ‘newCellDataSet’ function. Following this, genes were filtered in line with recommended parameters suitable for downstream analysis. For dimension reduction, we utilised the ‘reduceDimension’ function, applying the parameters ‘reduction_method = DDRTree’ and ‘max_components = 2’. This step is crucial for effectively simplifying the high‐dimensional data into a more manageable form. Subsequently, the cells were organised in a pseudotime sequence using the ‘orderCells’ function and visually represented with the ‘plot_cell_trajectory’ function. This approach allowed us to observe the developmental trajectory of cells across the pseudotime continuum. Furthermore, to visualise changes in the expression levels of biomarkers specific to different CAF subtypes along the pseudotime, we employed the ‘plot_genes_branched_pseudotime’ function.

### 2.4 Cell–cell communication network inference

To analyse cell–cell interactions between CAFs and other cellular categorises in the TME, we utilised the R package ‘CellChat’ (version 1.1.1).^23^ Our methodology, based on the ‘Secreted Signalling’ category from the ligand–receptor pairs database, was structured as follows: first, we calculated communication probability and inferred the communication network using ‘computeCommunProb’ and ‘filterCommunication’ functions. Second, this probability was determined at the signalling pathway level with the ‘computeCommunProbPathway’ function. Third, the comprehensive cell–cell communication network was ascertained using ‘aggregateNet’. We then selected specific signalling pathways for visualisation as heatmaps, employing the ‘netVisual_heatmap’ function.

### 2.5 Transcriptional regulatory network inference

The analysis of transcription factor (TF) regulatory networks at the single‐cell level was conducted using the Single‐Cell Regulatory Network Inference and Clustering (SCENIC) package within R (version 1.2.4),^24^ adhering to the default settings. For this purpose, a TF database aligned with the hg38 genome was chosen. This database, focusing on a search space of 10 kb around the transcription start site, was acquired using RcisTarget (version 1.10.0). Subsequently, gene regulatory networks were deduced using GENIE3 (version 1.12.0), based on gene expression data from CAFs. The distribution patterns of the enriched TFs were then depicted through plots utilising t‐distributed stochastic neighbour embedding methodology.

### 2.6 Multiplex immunofluorescence staining

For multiplex immunofluorescence (mIF) staining, we employed the Opal 7‐Color Manual IHC Kit (NEL811001KT, Akoya Biosciences). Formalin‐fixed, paraffin‐embedded tumour microarrays (TMAs) from STAD, LUAD, BRCA and COAD were selected for this staining process. The procedure began with the sequential application of primary antibodies targeting various CAF markers. These included the proCAF marker OGN (Osteoglycin, HPA013132, Sigma), myCAF marker α‐SMA (ab5694, Abcam), iCAF marker CCL2 (HPA019163, Sigma) and matCAF marker COL10A1 (12‐9771‐82, Invitrogen). Each antibody was used in a separate round of staining. Following the incubation with primary antibodies, fluorescently conjugated secondary antibodies from the Opal™ 7‐Color Manual IHC Kit were applied. This allowed for the visualisation of the bound primary antibodies under a fluorescence microscope. The immunofluorescence signals were captured using the Mantra™ Quantitative Pathology Imaging System. This system is equipped for high‐resolution multispectral imaging and analysis, enabling the simultaneous detection of multiple markers in a single tissue section. Such a capability is essential for comprehensively understanding the complex CAF landscape within the TME.

The quantification of fluorescence was conducted using inForm software, which utilises deep learning algorithms. The software develops a predictive model based on 20 typical mIF images, ensuring that the area under curve (AUC) remains above 85%. It is capable of recognising cell morphology, distinguishing CAFs and automating image analysis based on different biomarker staining conditions. Consequently, the software precisely quantifies different CAF subtypes in tissue sections. By integrating high‐throughput imaging with quantitative image analysis, we established a detailed and accurate method for evaluating the spatial distribution and density of various CAF subtypes within the TME. This approach enhances our understanding of the role of CAFs in cancer and their potential as therapeutic targets.

### 2.7 Immunohistochemistry staining and scoring

Immunohistochemistry (IHC) staining was conducted on STAD tissue microarray sections, utilising primary antibodies for OGN and ACTA2, each at a 1:100 dilution. For chromogen development, essential for visualising these proteins in the tissue sections, we used the EnVision system (Dako). The scoring criteria for IHC staining followed our previously established protocols, as detailed in an earlier report.^25^ Briefly, staining intensity was scored on a scale of 0–3, with 0 indicating no staining, 1 weak staining, 2 moderate staining and 3 strong staining. The extent of staining was scored based on the percentage of positively stained cells: 0 for no staining, 1 for 1%–25% of cells, 2 for 26%–50% of cells, 3 for 51%–75% of cells and 4 for 76%–100% of cells.

### 2.8 Establishment of a 3D co‐culture system of NF or CAF cell lines and STAD patient‐derived organoids

Patient‐derived organoids were established following a previously reported protocol,^26^ which involved the mechanical and enzymatic dissection of primary tumour tissues from gastric cancer patients, which were used to obtain single‐cell suspensions. These suspensions were subsequently encapsulated within Matrigel (Corning Incorporated) and preserved in an organoid medium. Organoids were propagated every 10 days and were employed for co‐culture experiments at passages 5. To establish the three‐dimensional (3D) co‐culture system, the organoids were collected and mechanically dissociated into smaller clusters. These organoid clusters were then embedded in Matrigel and preserved in organoid medium. On the following day, the NF or matCAF cell lines were uniformly seeded on the surface of the medium. The growth of organoids and CAFs was periodically monitored using a light microscope. After 15 days, observable differences in size and morphology among the organoid tissues were systematically documented.

### 2.9 Animal experiments

To explore the pro‐carcinogenic roles of matCAF and assess the effectiveness of the procyanidin C1 (PCC1) drug in vivo, we conducted a series of experiments involving NOD‐SCID gamma (NSG) mice. In the study of matCAF's pro‐carcinogenic effects, we divided the mice into two groups: a shRNA control group (shCtrl group) and a group in which the COL10A1 gene in matCAF was knocked down (shCOL10A1 group). We then employed two different injection models. In the first model, we combined breast cancer cell line MCF7 (10^6^ cells/mouse) with matCAF cells (10^6^ cells/mouse) in a 1:1 ratio, mixed with matrix gel, and administered subcutaneous injections (*n* = 8 per group). After 20 days, we documented the tumours through photography and determined their weights. In the second model, gastric cancer luc‐NCI‐N87 cells (10^6^ cells/mouse) were similarly combined with matCAF cells (10^6^ cells/mouse) in a 1:1 ratio with matrix gel and administered as intraperitoneal (i.p.) injections (*n* = 5 per group). After 15 days, we captured images of the animals using an in vivo fluorescence imaging system. Notably, we quantified the digested cells using blood counting plates. Furthermore, based on preliminary experiments, we established that the optimal ratio of cancer cells to matCAF is 1:1, as it allows for the development of observable tumours within a manageable timeframe.

To evaluate the in vivo efficacy of the PCC1 drug, we mixed MNK45 cells (10^6^ cells/mouse) with gastric cancer patient‐derived CAFs (10^6^ cells/mouse) in a 1:1 ratio with matrix gel and subsequently administered subcutaneous injections into the dorsal flank of 4‐week‐old NSG mice. Three days post‐injection, we randomly divided the mice into two groups: (i) the vehicle group (*n* = 10), which received i.p. injections of a vehicle solution (20% PEG300 and 2% Tween‐80 in saline), and (ii) the PCC1 group (*n* = 10), which received i.p. injections of 20 mg/kg PCC1 (HY‐N2342, MedChemExpress) once daily. The drug concentrations used in the mouse experiments were based on previous studies.^27^ After 20 days of drug administration, we euthanised the mice, extracted the tumour tissues, weighed them and fixed them for mIF staining. Pathology sections stained with mIF were imaged using the Mantra Quantitative Pathology Imaging System. For each section, we analysed four randomly selected fields of view to gather data on matCAF using inform software.

All procedures involving animal handling and experimentation were conducted with the approval of the Hong Kong Department of Health and the Animal Experimentation Ethics Committee of the Chinese University of Hong Kong (AEEA Ref. No.: 21‐013‐NSF).

### 2.10 Statistical analyses

Our statistical analyses were executed using R software (version 4.0.2), GraphPad Prism (version 8.0.1) from GraphPad Software and SPSS software (version 22.0) from IBM Corp. For comparing tumour weight and cell density among groups, we employed the Student's *t*‐test. The Cox regression model was utilised to analyse the survival rate for each parameter. In instances where variables demonstrated statistical significance in univariate survival analysis, a multivariate survival analysis was conducted using the Cox proportional hazards model, incorporating likelihood ratio statistics. All *p*‐values mentioned are two‐tailed, with values under .05 regarded as statistically significant. Values less than .001 were considered highly significant, and those under .0001 were viewed as extremely significant.

## 3 RESULTS

### 3.1 Construction and analysis of a pan‐cancer CAF atlas: a comprehensive workflow

Our research initiated with the creation of the pan‐cancer CAF atlas, which is derived from scRNA‐seq data of nearly 500 000 cells across 210 patients, spanning 12 distinct cancer types. This carefully compiled dataset includes various cancers, such as BRCA,^28^ with analysis on 27 890 cells from five patients, and cholangiocarcinoma (CHOL),^29^ encompassing a study of 40 026 cells from 10 patients. COAD^30^ involved 54 432 cells from 33 patients, while STAD^31^ included 38 390 cells from 22 patients. Hepatocellular carcinoma (LIHC)^32^ saw an examination of 31 235 cells from seven patients, and LUAD^33^ contributed a significant dataset of 207 876 cells from 58 patients. Further, neuroendocrine prostate cancer (NEPC)^34^ data came from 20 962 cells across six patients, and pancreatic ductal adenocarcinoma (PAAD)^35^ involved a detailed study of 96 063 cells from 35 patients. Prostate adenocarcinoma (PRAD)^36^ research spanned 35 659 cells from 13 patients, Uterine corpus endometrial cancer (UCEC)^37^ investigated 39 101 cells from six patients, ovarian cancer (OV)^37^ included 27 764 cells from four patients, and uveal melanoma (UVM)^38^ provided data from 59 915 cells across 11 patients. In terms of cancer subtypes, we scrutinised BRCA samples, categorising them into three distinct subtypes: estrogen receptor‐positive (ER^+^), progesterone receptor‐positive (PR^+^) and triple‐negative breast cancer, each presenting unique biological characteristics. Beyond BRCA, our investigation extended to a variety of other cancers. These included CHOL, specifically intrahepatic cholangiocarcinoma; STAD, COAD and LIHC, focusing on primary colorectal cancer; LUAD, under the broader category of non‐small cell lung cancer; NEPC, particularly the castration‐resistant subtype; PAAD, concentrating on primary pancreatic ductal adenocarcinoma; PRAD, specifically primary prostate cancer; OV, including endometrioid and serous types; UCEC focusing on endometrioid and serous endometrial cancer; and UVM, encompassing uveal melanoma of mixed cell type. This extensive evaluation elucidates the heterogeneity within and across various cancer types, offering critical insights for targeted therapeutic strategies. This comprehensive analysis illuminates the heterogeneity within and across various cancer types, providing essential insights for targeted therapeutic approaches.

**FIGURE 1:**
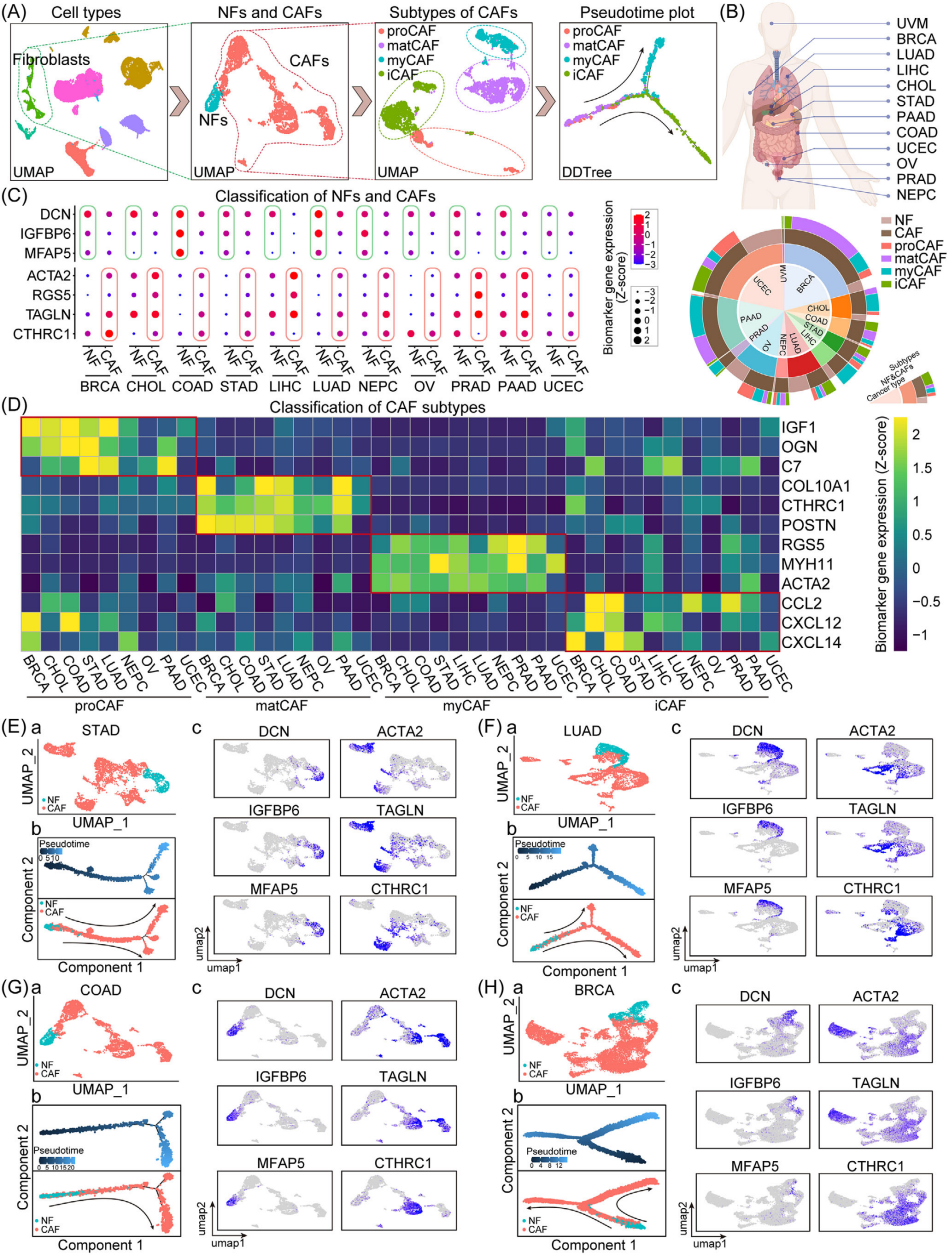
Comprehensive classification of fibroblasts through pan‐cancer single‐cell RNA sequencing (scRNA‐seq) analysis. (A) Schematic representation of the single‐cell transcriptomic analysis process employed to identify cancer‐associated fibroblast (CAF) subtypes. Fibroblasts were separated from other cell types within each sample, and then categorised into normal fibroblasts (NFs) and CAFs. Subsequently, based on distinctive molecular attributes, CAFs were further partitioned into progenitor CAF (proCAF), matrix‐producing CAF (matCAF), myofibroblastic CAF (myCAF) and inflammatory CAF (iCAF) subtypes. Pseudotemporal analyses were executed to elucidate the developmental trajectories of these subtypes. (B) Pie chart illustrating the distribution of NFs and various CAF subtypes across 12 types of cancer: uveal melanoma (UVM), breast cancer (BRCA), lung cancer (LUAD), hepatocellular carcinoma (LIHC), cholangiocarcinoma (CHOL), gastric cancer (STAD), pancreatic ductal adenocarcinoma (PAAD), colorectal cancer (COAD), uterine corpus endometrial cancer (UCEC), ovarian cancer (OV), Prostate adenocarcinoma (PRAD) and neuroendocrine prostate cancer (NEPC). (C) The classification of fibroblasts in this study relied on several biomarkers: *DCN*, *IGFBP6* and *MFAP5* (for NFs), and *ACTA2*, *RGS5*, *TAGLN* and *CTHRC1* (for CAFs). The expression of these biomarkers among NFs and CAFs underscores the accuracy of our single‐cell subgrouping. (D) The identification of four CAF subtypes was facilitated by their respective subtype‐specific biomarkers: *IGF1*, *OGN* and *C7* for proCAFs; *COL10A1*, *CTHRC1* and *POSTN* for matCAFs; *RGS5*, *MYH11* and *ACTA2* for myCAFs; *CCL2*, *CXCL12* and *CXCL14* for iCAFs. (E–H) Detailed classification of NFs and CAFs in STAD, LUAD, COAD and BRCA is showcased via uniform manifold approximation and projection (UMAP) plots, pseudotemporal analysis plots, and feature plots of biomarker expression. For each cancer type, a UMAP plot (a) separates NFs and CAFs within the fibroblast population, a pseudotemporal analysis (b) reveals the developmental trajectory of CAFs, and a feature plot (c) illustrates the expression distribution of NF and CAF biomarkers.

Each dataset underwent standard single‐cell data analysis procedures, in terms of filtering, dimension reduction and clustering. This method was key in identifying fibroblasts by their specific biomarkers and subsequently segregating them from other cell types in the dataset using appropriate gene expression thresholds. Following this, fibroblasts were categorised into two primary groups: NFs and CAFs. Remarkably, CAFs presented a diverse spectrum, further divisible into four distinct subtypes: proCAF, matCAF, myCAF and iCAF. After establishing these classifications, we conducted pseudotime analysis, which revealed the developing evolution of these CAF subtypes (Figure 1A). A notable finding from our multidimensional pie chart analysis was the significant diversity in the prevalence of CAF subgroups among various cancer types. To illustrate, a substantial presence of matCAF was observed in breast cancer (BRCA), while this subtype was entirely absent in LIHC (Figure 1B). Notably, the breast cancer samples analysed were exclusively from late‐stage tumours. On the other hand, the hepatocellular carcinoma (HCC) samples were from early‐stage tumours, characterised by minimal scarring and having METAVIR scores ranging from F1 to F2. It underscores that the matCAF population is more prevalent in the advanced phases of cancer, thereby suggesting a potential contribution to the tumour progression. Meanwhile, myCAF was the predominant subtype of CAF in LIHC samples. Previous studies corroborate this observation, revealing that over 80% of HCCs develop in livers affected by fibrosis or cirrhosis.^39^ Indeed, α‐SMA in hepatic fibrosis is not only a marker for identifying activated myofibroblasts, but also for the degree of fibrosis.

The clustering of fibroblasts was anchored on several key biomarkers. NFs were identified using established markers such as DCN, IGFBP6 and MFAP5,^40^, ^41^, ^42^ while CAFs were distinguished using biomarkers such as ACTA2, RGS5, TAGLN and CTHRC1^43^, ^44^, ^45^, ^46^ (Figure 1C). In STAD, LUAD, COAD and BRCA, we consistently identified fibroblasts that had transitioned from NFs, as indicated by their high expression of these CAF biomarkers. This enabled us to successfully isolate and define these CAFs (Figures 1E–H and S1). Further isolation and in‐depth analysis of these CAFs led to the identification of four unique molecular CAF subtypes. Each subtype was characterised by its notably elevated levels of certain genes within the cluster (Figure 1D). The significant discovery shall be elaborated in subsequent sections of our study. To promote data sharing and facilitate further research, we have developed a dedicated website for this study, available at http://www.cafatlas.com/. This platform serves as a comprehensive and accessible resource for the global research community. It allows for the exploration, comparison, and understanding of the intricate complexities of CAFs across various cancer types, enhancing collaborative efforts in cancer research.

### 3.2 Identification and characterisation of distinct biomarkers within four CAF subtypes

**FIGURE 2:**
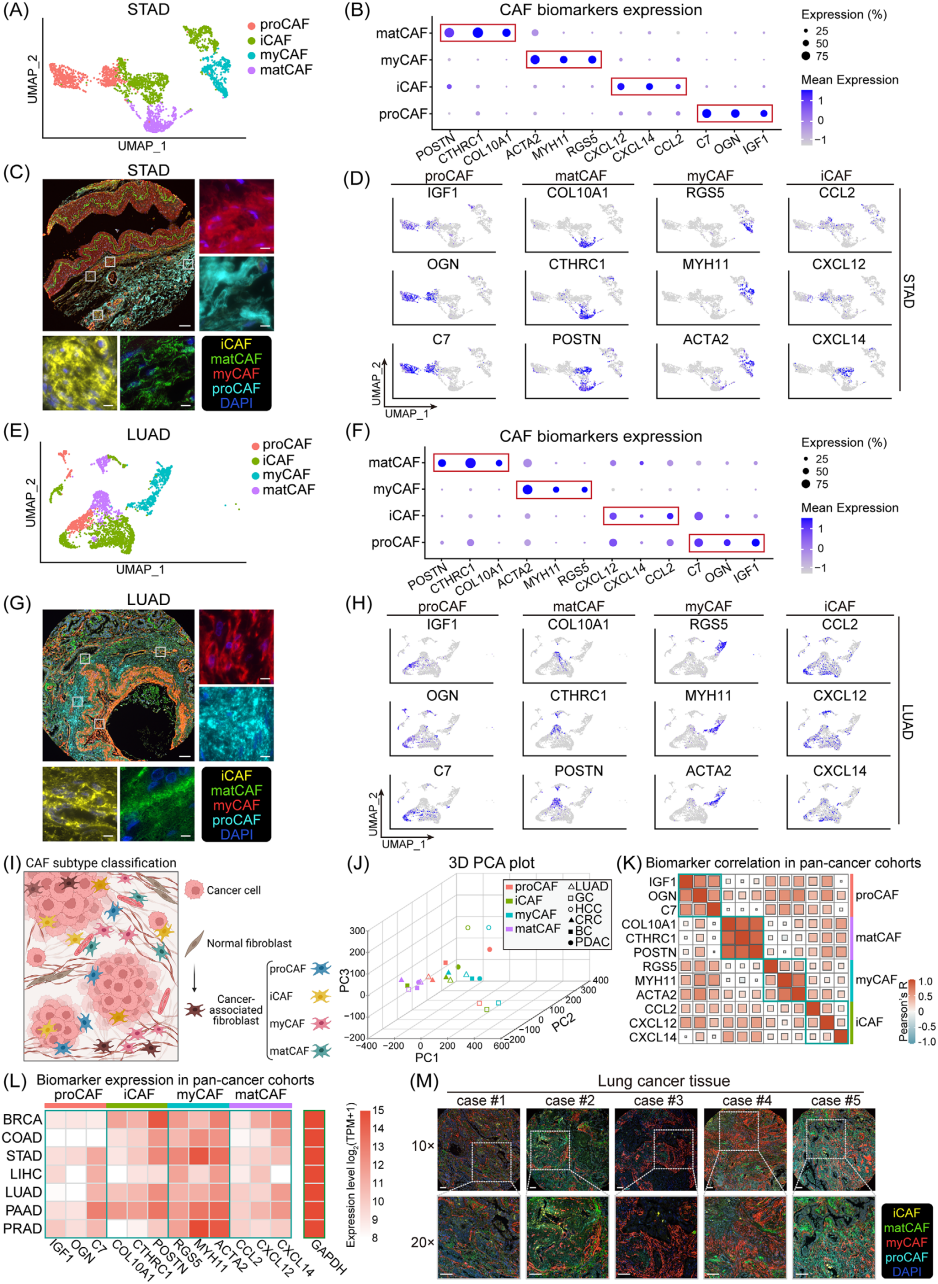
Characterisation and biomarker expression of four cancer‐associated fibroblast (CAF) subtypes. (A) Uniform manifold approximation and projection (UMAP) plot demonstrating the classification of four CAF subtypes in STAD. (B) Bubble heatmap illustrates the expression levels of subtype‐specific biomarkers in STAD, highlighting elevated proportions and average expression levels of respective biomarkers within corresponding CAF subtypes. (C) Representative multiplex immunofluorescence (mIF) images of gastric cancer tissues from STAD patients, stained with DAPI (blue), anti‐COL10A1 (green), CCL2 (yellow), OGN (cyan) and α‐smooth muscle actin (α‐SMA) (red) antibodies. Large image taken at 10× magnification (scale bar = 200 μm), with an inset of the same tissue captured at 40× magnification (scale bar = 50 μm). (D) UMAP plots depict biomarker expression levels across all CAF subtypes in STAD, demonstrating enhanced expression within corresponding CAF subtype populations. (E–H) Similar CAF subtype distribution and biomarker expression patterns observed in LUAD as in STAD. (I) Schematic overview of CAF subtypes illustrating spatial distribution of each subtype. (J) Three‐dimensional principal component analysis (PCA) plot showcases clustering of CAF subtypes across various solid tumours. (K) Within each CAF subtype, specific markers exhibit a positive correlation. (L) Expression levels and relative abundance of biomarkers analysed in The Cancer Genome Atlas (TCGA) cohort. (M) Representative mIF images of tissue microarrays from LUAD. DAPI (nucleus) is shown in blue, COL10A1 (matrix‐producing CAF [matCAF]) in green, CCL2 (inflammatory CAF [iCAF]) in yellow, OGN (progenitor CAF [proCAF]) in cyan and α‐SMA (myofibroblastic CAF [myCAF]) in red. Upper image from each of the five cases was taken at 10× magnification, with the corresponding lower image captured at 20× magnification from the same tissue (outlined regions in the 10× image correspond to the areas shown in the 20× image), and all scale bars represent 200 μm. DAPI, 4',6‐diamidino‐2‐phenylindole.

In our effort to elucidate the heterogeneity within CAFs, we performed unsupervised clustering following the isolation of CAFs from their NF counterparts. This stratification process led to the identification of four distinct CAF subtypes: proCAF, iCAF, myCAF and matCAF. These subtypes were distinguished based on the relative abundance and expression levels of unique biomarkers. The distribution of these CAF subtypes, along with their specific biomarkers across different cancers, is comprehensively illustrated in Figures 2A–H and S2–S5. Each subtype was characterised by a set of biomarkers with notably high gene expression frequencies. The proCAF subtype predominantly expressed genes such as IGF1, OGN and C7. The matCAF subtype was marked by elevated levels of COL10A1, CTHRC1 and POSTN, while the myCAF subtype showed high expression of RGS5, MYH11 and ACTA2. The iCAF subtype was identified by increased levels of CCL2, CXCL12 and CXCL14. We found that LIHC and PRAD mainly consisted of myCAFs, with a limited presence of iCAFs. Notably, matCAF and proCAF subtypes were predominantly missing from these types of cancers, possibly due to insufficient sample sizes.

Interestingly, on the UMAP, proCAF and iCAF appeared close to each other, as did matCAF and myCAF, despite their biomarkers exhibiting specificity. This proximity suggests gene expression similarities between proCAF and iCAF, and a similar pattern is observed between matCAF and myCAF. To further validate the independent existence of each CAF subtype within the TME, we employed mIF staining on TMA samples from patients with STAD, LUAD and COAD. This staining revealed distinct spatial distributions of CAF subtypes within the same tissue samples (Figures 2C,G,M and S5 and Tables S1–S4). Notably, iCAFs were found interspersed throughout the tumour mesenchyme, often localising near blood vessels, implying a potential role in angiogenesis or vasculature‐related functions. On the other hand, myCAF and matCAF subtypes showed a more clustered distribution, suggesting possible synergistic interactions in their functional roles. Additionally, our research expanded to include further analysis of additional CAF biomarkers such as *S100A4*, *PDPN* and *FAP*. Upon examination through single‐cell UMAP visualisations, it became evident that the expression patterns of these genes did not demonstrate significant specificity for effectively distinguishing between the various CAF subgroups (Figure S6). This lack of distinct expression profiles across the subgroups suggests that while these markers are present, they may not serve as reliable indicators for clear subgroup classification within the CAF spectrum. Additionally, platelet‐derived growth factor receptor‐beta (PDGFR‐β) was initially regarded as a CAF marker,^47^ while lacked the necessary specificity for classifying CAF subtypes effectively. Consequently, we decided not to use PDGFR‐β as a distinct marker in our subtype classification.

Additionally, 3D PCA plots at the single‐cell level revealed a significant finding: matCAF clusters were consistent across all cancer types, suggesting a highly stable genetic signature of matCAF in solid tumours (Figure 2J). Additionally, the expression levels of markers specific to the same CAF subtype showed a high positive correlation, particularly within matCAF (Figure 2K). These finding challenges previous assumptions and confirms matCAF as a distinct and defined entity.

To validate the reliability of these biomarkers, we analysed The Cancer Genome Atlas (TCGA) pan‐cancer cohort. We normalised gene expression levels across different cancer types using the housekeeping gene GAPDH. The results showed that these genes exhibited elevated and measurable expression levels, meeting the criteria for effective biomarkers. Furthermore, the expression levels of these biomarkers demonstrated consistency across various cancer types (Figure 2L). Moreover, Figures 2 and S2–S5 comprehensively illustrate and provide supporting evidence for the characterisation, spatial distribution and unique expression patterns of biomarkers across the four identified CAF subtypes. This extensive analysis has greatly enhanced our understanding of CAF heterogeneity. It marks a significant step forward in developing more precise and personalised therapeutic strategies for cancer treatment, tailoring interventions to the specific CAF subtypes present within the TME.

### 3.3 Activation diversity among transcription factors of CAF subtypes

**FIGURE 3:**
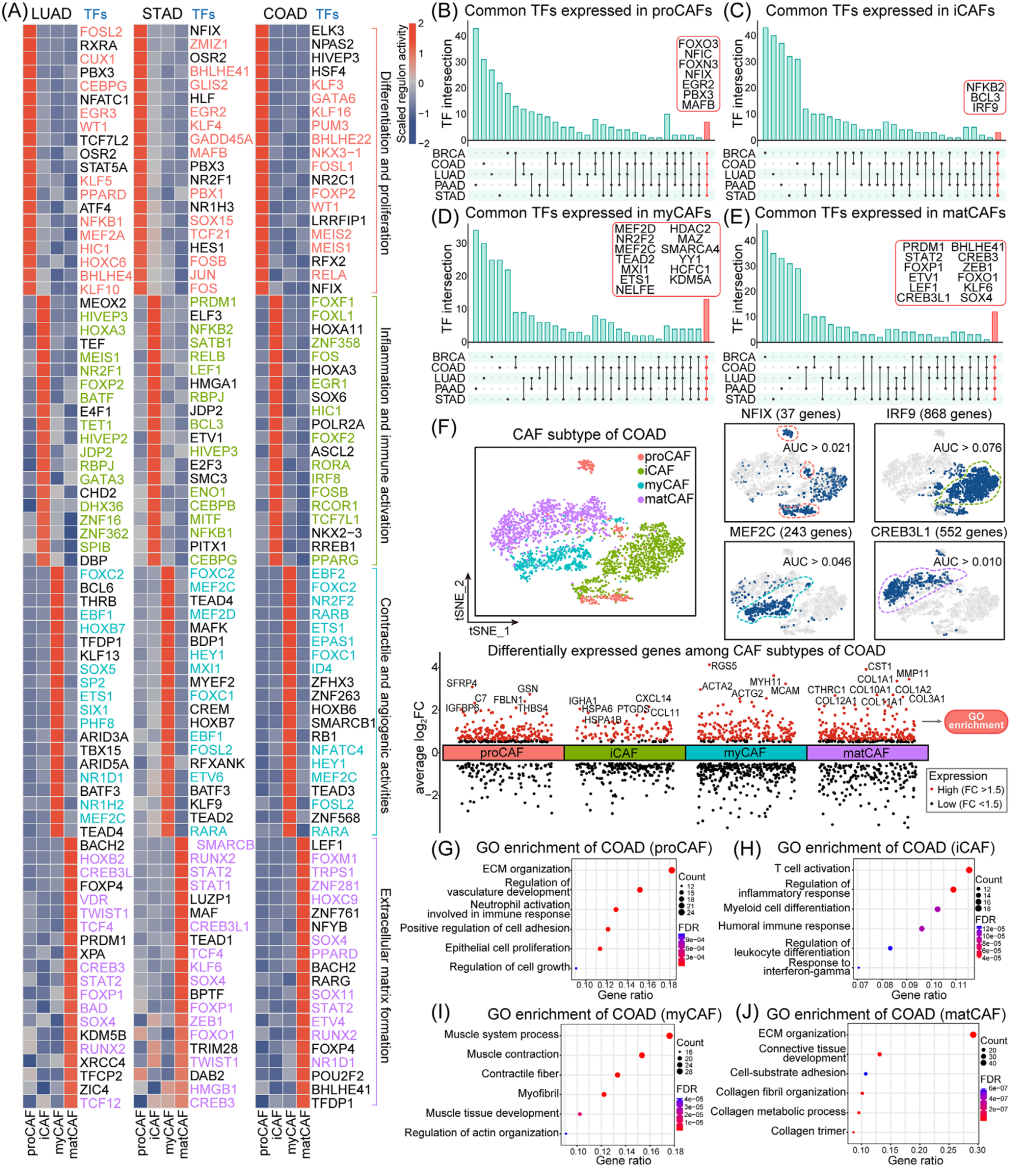
Distinguishing functions among cancer‐associated fibroblast (CAF) subtypes. (A) The activation levels of the top 20 transcription factors (TFs) for each CAF subtype in LUAD, STAD and COAD are displayed. TFs with functions corresponding to the CAF subtypes are highlighted. (B) The top 100 enriched TFs for progenitor CAFs (proCAFs) in BRCA, COAD, LUAD, PAAD and STAD were identified. The intersection plot presents the number of shared TFs across different combinations of cancers. TFs enriched across all cancer types are indicated within the red box. (C–E) Analogous analyses were also performed for inflammatory CAF (iCAF) (C), myofibroblastic CAF (myCAF) (D) and matrix‐producing CAF (matCAF) (E). (F) A t‐distributed stochastic neighbour embedding (t‐SNE) plot illustrates the CAF classification in COAD, using four TFs (*NFIX*, *IRF9*, *MEF2C* and *CREB3L1*) as examples of enriched TFs in proCAF, iCAF, myCAF and matCAF, respectively (the number of genes in each TF's regulon is noted in brackets). Cells with area under curve (AUC) scores exceeding the threshold are highlighted and align with the corresponding CAF subtype population as indicated by the t‐SNE plot. (G) A volcano plot shows the specific high expression of genes among different CAF subtypes in COAD. Differentially expressed gene (DEG) analysis was conducted between each CAF subtype and the others. Notably, genes with a log_2_|fold change| > 1.5 and *p* < .05 are coloured in red and subjected to subsequent Gene Ontology (GO) enrichment analysis. (H–K) The GO enrichment plot reveals that proCAFs are involved in proliferation, as well as functions carried out by other CAF subtypes in COAD (H). Genes with high expression related to immune responses are enriched in iCAFs (I). Furthermore, genes implicated in contractile and muscle‐related events are enriched in myCAFs (J), whereas genes controlling extracellular matrix (ECM) formation are enriched in matCAFs (K).

Upon distinguishing four distinct CAF clusters, our attention turned to deciphering their distinct roles. With the understanding that transcriptional regulatory networks often control cellular functions, we applied the SCENIC methodology to identify key TFs across the pan‐cancer single‐cell gene expression dataset.^24^, ^48^ This approach facilitated the identification of the 20 most prominently active TFs for every CAF subtype in LUAD, STAD, COAD and BRCA (Figures 3A and S7A). Our analysis revealed a notable pattern: the most active TFs within identical CAF categories often showed functional similarities. This finding strongly supports the hypothesis that TF expression and control differ between CAF categories, suggesting that variances in functional roles among these subtypes may be linked to variations in TF expression.

To delve deeper into this pattern, we conducted a thorough analysis across different forms of cancer. We first pinpointed the 100 most prevalent TFs for every CAF subtype within each form of cancer. We then intersected these lists to identify TFs consistently enriched across all cancer types (Figure 3B–E). In this analysis, the intensity of cell colouration indicated the level of TF activity within the core regulon network, with darker cells representing higher activity. For instance, in COAD, we noted that NFIX, a TF associated with cellular growth, was predominantly present in proCAF.^49^ In contrast, *IRF9*, a TF involved in immune response regulation, was primarily found in iCAF,^50^ while *MEF2C*, influencing myogenesis and angiogenesis, was mostly present in myCAF.^51^ Lastly, *CREB3L1*, a TF that regulates collagen formation,^52^ was uniquely expressed in matCAF (Figure 3F). Interestingly, similar patterns were noted in other cancer types such as BRCA and LUAD, where the active TF regulatory networks of diverse CAF subtypes likewise demonstrated significant functional distinctions (Figure S7B,C). These observations reinforce our understanding that the functionality of various CAF subtypes differs within tumour tissues, thereby underscoring their heterogeneous nature and potential impact on tumour progression and treatment response.

### 3.4 Identification of specifically highly expressed genes and functional enrichment through Gene Ontology analysis

To further characterise the capabilities of different CAF subtypes, the possibility has been considered that highly expressed genes might hint at specific cellular functions. Therefore, we identified genes which were highly abundant in each CAF subgroup and then conducted a Gene Ontology (GO) functional enrichment analysis.

Interestingly, as depicted in Figure 3G, the genes predominantly expressed in the matCAF subtype in COAD were primarily members of the collagen family, marking a stark contrast to the other three CAF subtypes. Further enrichment analysis revealed distinct functional roles for each CAF subtype: the iCAF was involved in orchestrating immune responses; the myCAF was associated with contractile activities; and the matCAF in charge of ECM reformation (Figures 3H–K and S8A–D). These results align with those obtained from the SCENIC analysis, supporting our hypothesis that CAF subtype‐specific transcription factor expression may drive the observed functional heterogeneity among CAFs.

For additional validation of these GO enrichment pathways, we conducted single‐cell gene set enrichment analysis for each CAF subtype, calculating the AUC scores at a single‐cell level. Both STAD and LUAD exhibited high activation in their respective CAF subtypes related to stem cell maintenance, adaptive immune responses, myofilament events and collagen modifications (Figure S8E,F). These findings reinforce our earlier observations. Moreover, the proCAF subtype was found to share functions with different CAF categories, besides its unique growth‐related activities. This suggests that proCAF might serve as a foundational source for various CAF categories, aligning with multiple research that propose CAF differentiation and role divergence in the TME.^3^, ^8^

### 3.5 Evolutionary pathway of CAF subtypes in tumour advancement

**FIGURE 4:**
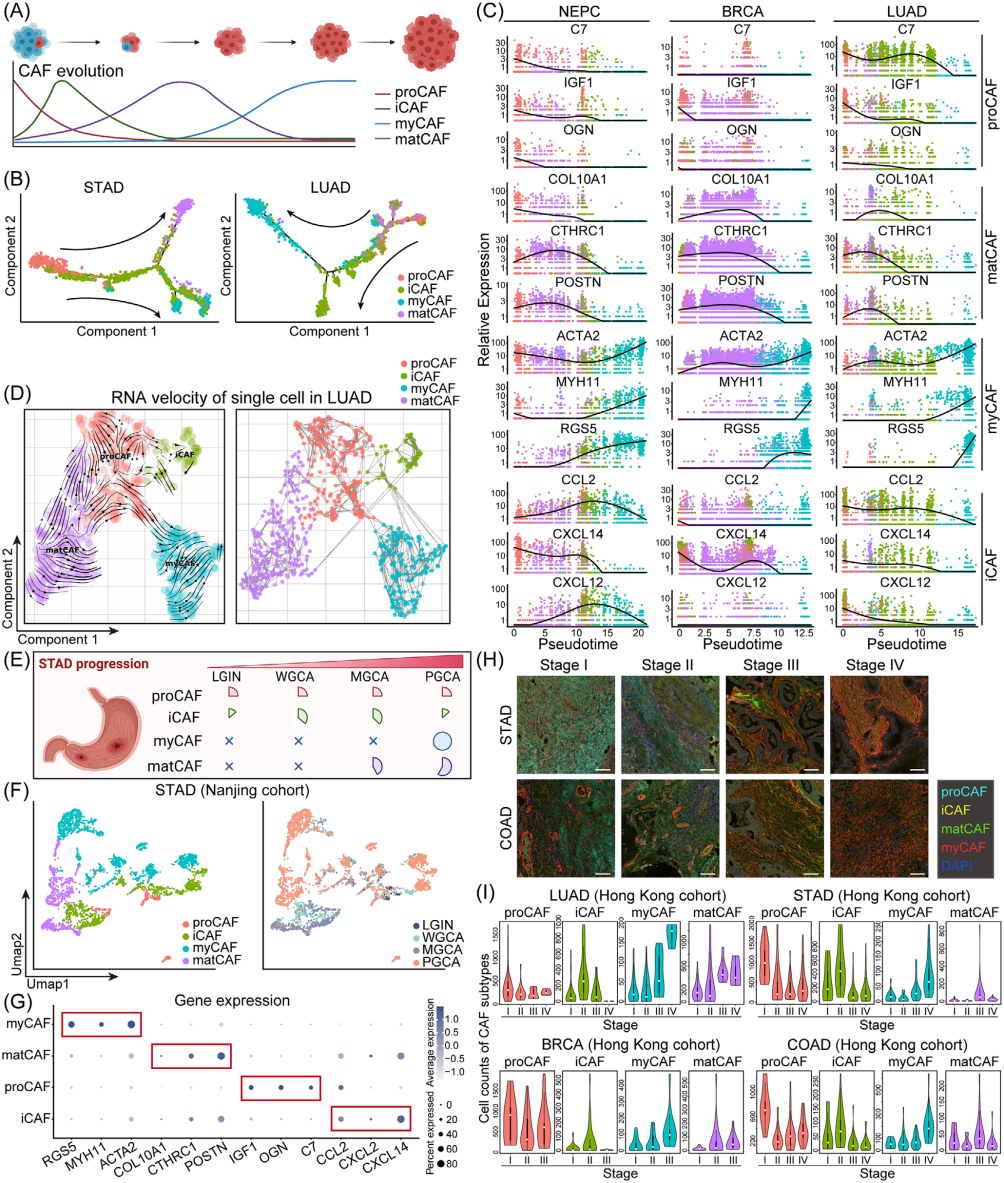
Depiction of the typical evolutionary trajectories of cancer‐associated fibroblast (CAF) subtypes in solid tumours. (A) A schematic illustrating the evolution of CAF subtypes throughout tumour progression. (B) The arrangement of CAF subtypes along pseudotimes within the two‐dimensional state space as determined by Monocle2. This pseudotemporal analysis indicates that progenitor CAF (proCAF) functions as the developmental progenitor of the other CAF subtypes in both STAD and LUAD. (C) The developmental trajectories of CAF subtypes in neuroendocrine prostate cancer (NEPC), BRCA and LUAD are inferred through a single‐cell pseudotime analysis based on the expression levels of CAF subtype biomarkers. (D) A similar analysis at the single‐cell RNA splicing level, where RNA velocity maps depict the evolution of CAF subtypes from proCAF to myofibroblastic CAF (myCAF). (E) A schematic representation detailing the dynamic shifts in the distribution of different CAF subtypes in STAD alongside cancer progression, using an in‐house single‐cell RNA sequencing (scRNA‐seq) dataset. (F) Within the Nanjing STAD cohort, single‐cell uniform manifold approximation and projection (UMAP) plots of different CAF subpopulations are presented in relation to various stages of disease progression. (G) A bubble heatmap exhibits the expression levels of subtype‐specific biomarkers in STAD, accentuating the increased proportions and average expression levels of respective biomarkers within corresponding CAF subtypes. (H) Representative multiplex immunofluorescence (mIF) images of STAD and COAD highlight the expression changes of biomarkers for different CAF subtypes (scale bar = 200 μm). (I) Violin plots elucidate the changes in cell counts of the four CAF subtypes across tumour stages in LUAD, STAD, BRCA and COAD.

Our exploration into the maturation dynamics of CAFs included an analysis of the developmental interconnections between CAF subtypes through single‐cell pseudotime analysis. In this pseudo‐chronological framework, proCAF consistently emerged as the initial population, justifying its designation as the ‘progenitor’. myCAF invariably emerged at the final phase of this developmental sequence, while matCAF and iCAF were identified in intermediate stages (Figures 4A,B, S2 and S3). Analysis of RNA expression intensity in correlation with pseudotime showed that proCAF biomarkers were prevalent at the beginning, whereas iCAF and matCAF biomarkers gradually increased in expression with tumour advancement. Conversely, myCAF biomarkers reached their peak towards the end, with minimal expression of the other three CAF subtypes' biomarkers at this stage (Figure 4C). We also employed RNA velocity estimation,^53^ distinguishing between unspliced and spliced mRNAs, in the lung cancer (LUAD)‐specific CAF scRNA‐seq dataset (Figure 4D). This analysis further supported the observation that CAF subtypes evolve towards myCAF.

Moreover, our in‐house scRNA‐seq dataset enabled us to trace the dynamic shift in the distribution of various CAF subtypes during STAD progression (Figure 4E). UMAP plots from the Nanjing STAD cohort illustrated the transformation of CAF subpopulations at different stages of cancer, with an increase in myCAF and matCAF as STAD advanced (Figure 4F). A bubble heatmap highlighted the expression levels of subtype‐specific biomarkers in STAD, revealing an increase in the proportion and average expression levels of each biomarker in its corresponding CAF subtype (Figure 4G). Furthermore, mIF images of STAD and COAD emphasised the shifts in biomarker expression for different CAF subtypes (Figure 4H and Tables S1 and S4). These changes were also depicted in violin plots, showing the cell counts of the four CAF subtypes across different tumour stages in LUAD, STAD, BRCA and COAD (Figure 4I and Tables S5–S8). Our observations reveal that proCAF and iCAF are predominant in early stages of cancer, whereas myCAF and matCAF become more prevalent in advanced stages. This indicates a dynamic pattern in the evolution of CAF subtypes during tumour progression, suggesting a complex interplay of plasticity and adaptability within the TME.

### 3.6 Intercommunication networks among distinct CAF subtypes in cellular context

**FIGURE 5:**
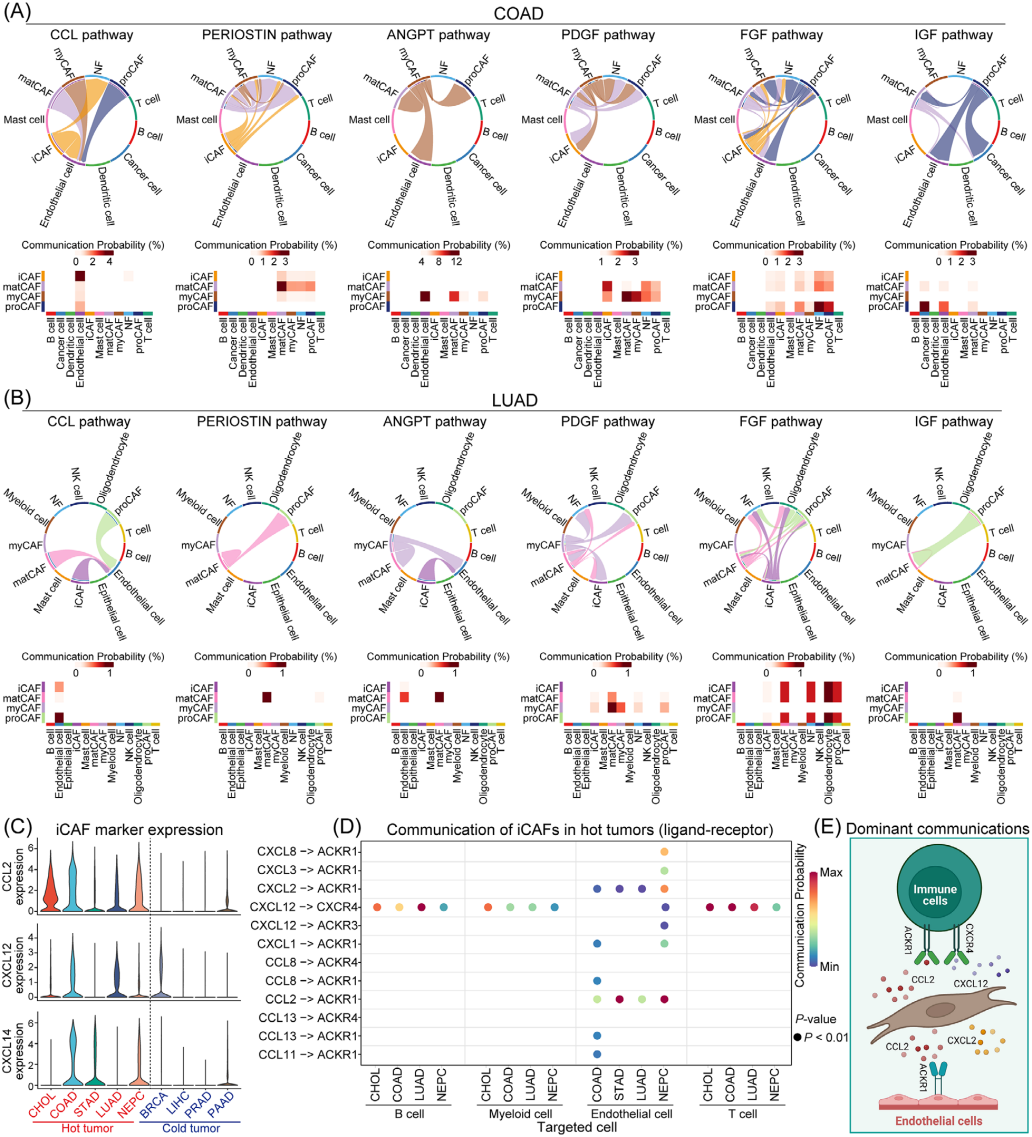
Detailed interactions of cancer‐associated fibroblast (CAF) subtypes with other cell types at the single‐cell level. (A) Essential interaction pathways between each CAF subtype and other cell types in COAD were determined, using communication probability as an indication of interaction strength. Chosen interaction pathways included the C–C motif chemokine ligand (CCL) pathway (inflammatory CAF [iCAF]), the PERIOSTIN pathway (matrix‐producing cancer‐associated fibroblast [matCAF]), the angiopoietin (ANGPT) and platelet‐derived growth factor (PDGF) pathways (myofibroblastic CAF [myCAF]), along with the fibroblast growth factor (FGF) and insulin‐like growth factor (IGF) pathways (progenitor CAF [proCAF]). (B) An analogous analysis was conducted for each CAF subtype in LUAD, yielding results consistent with those discerned in COAD. (C) The comparison of expression levels of iCAF biomarkers (*CCL2*, *CXCL12*, *CXCL14*) in hot versus cold tumours revealed heightened expression levels of all iCAF markers in hot tumours. (D) A schematic depiction of the iCAF interaction mechanism in hot tumours, where the CXCL12–CXCR4 ligand–receptor pair participated in interactions with B cells, myeloid cells and T cells. Concurrently, the CXCL2–ACKR1 and CCL2–ACKR1 ligand–receptor pairs contributed to interactions with endothelial cells. (E) This schematic representation illustrates the main ligand–receptor interactions between iCAFs and immune cells, and endothelial cells, showcasing the complexity of the communication within the tumour microenvironment (TME).

To delve deeper into the functional dynamics of CAFs, we examined their interactions with other cellular entities within the TME (Figures 5A,B, S9 and S10). Across various cancer types, a consistent pattern was observed. The iCAF subtype played a role in the C–C motif chemokine ligand (CCL) pathway, a crucial immune‐oriented pathway impacting endothelial cells. Meanwhile, the matCAF subtype engaged in the PERIOSTIN pathway, essential for ECM formation. The proCAF subtype was linked to the fibroblast growth factor and insulin‐like growth factor (IGF) pathways, vital for cell proliferation and development, affecting various cell types within the TME. Unexpectedly, the myCAF subtype was associated with the angiopoietin and platelet‐derived growth factor pathways, contributing to angiogenesis and primarily acting on endothelial cells and matCAF.

Previous studies have noted variation in iCAF expression across different cancer types.^5^ To understand this, we categorised cancers into ‘hot’ and ‘cold’ tumours. ‘Hot’ tumours typically have abundant tumour‐infiltrating lymphocytes and usually respond better to immunotherapy, while ‘cold’ tumours have sparse lymphocyte infiltration and generally show a poorer response to such treatments. Intriguingly, iCAF biomarkers were detected predominantly abundant in ‘hot’ tumours but were almost disappeared in ‘cold’ tumours, which may account for the resistance of ‘cold’ tumours to immunotherapy (Figure 5C). Our research delved deeper into the ‘iCAFs’ interactions with other cell types within the TME of ‘hot’ tumours. It was persistently observed that iCAFs were consistently found to interact with immune cells, such as B cells, myeloid cells and T cells, predominantly via the CXCL12‐CXCR4 ligand–receptor pairs. Additionally, iCAFs communicated most extensively with endothelial cells in solid tumours, primarily through the CCL2–ACKR1 and CXCL2–ACKR1 ligand–receptor pairs (Figure 5D). Figure 5E graphically represents these dominant communications between iCAFs and immune cells, as well as endothelial cells. In conclusion, our findings highlight the complex and dynamic network of interactions between CAF subtypes within the TME. These interactions not only deepen our understanding of how CAFs exert their functions but also point to their potential as targets for therapeutic intervention in cancer treatment.

### 3.7 Clinical implications of CAF subtypes as prognostic indicators

**FIGURE 6:**
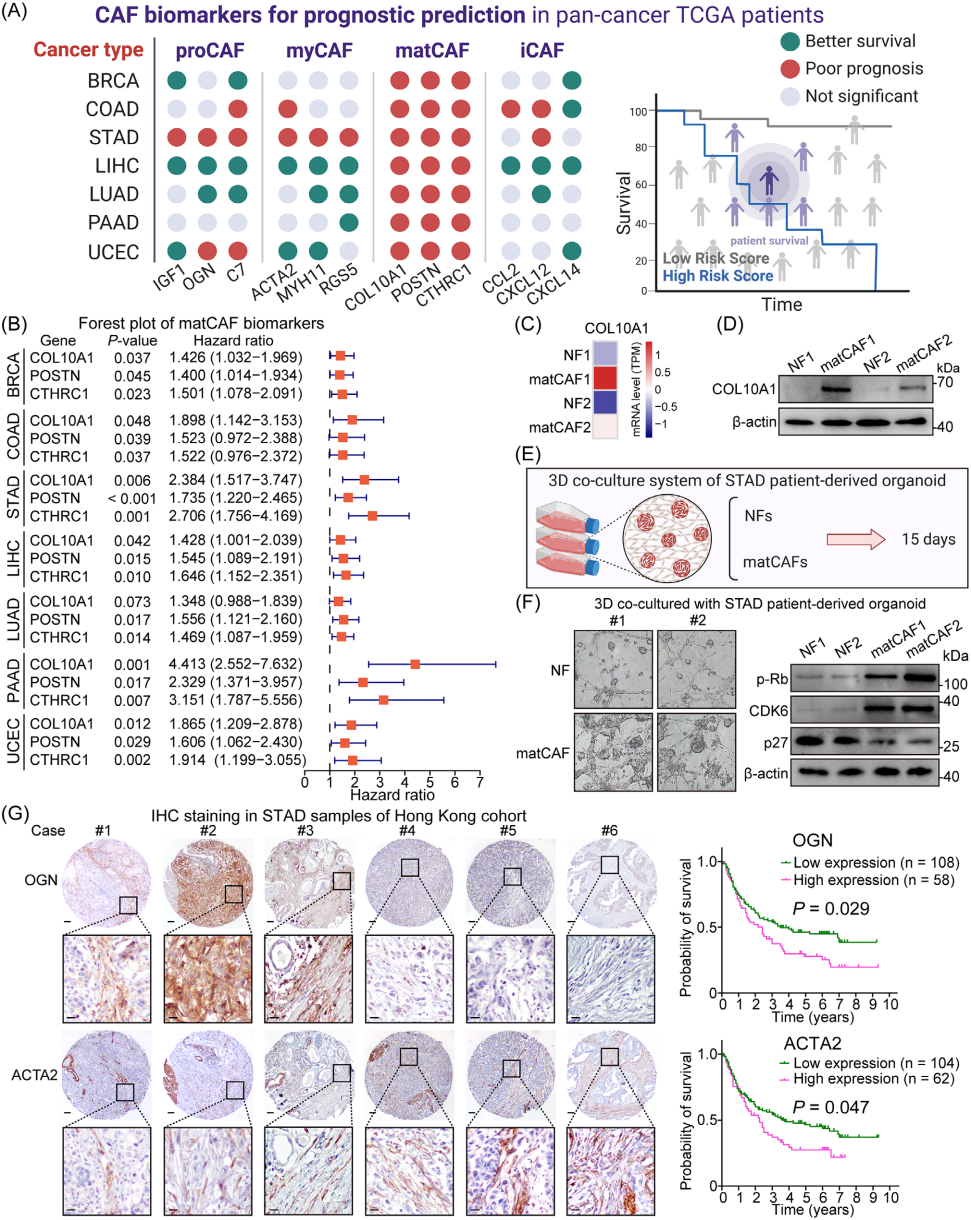
Clinical significance of different cancer‐associated fibroblast (CAF) subtypes in pan‐cancer patients. (A) A comprehensive graph indicating the prognostic relevance of CAF subtype biomarkers across several cancer types. Green colour indicates that highly expressed biomarkers are significantly associated with improved patient survival, while red colour signifies that highly expressed biomarkers predict a significantly worse prognosis for cancer patients. Grey colour indicates no statistical difference. (B) A forest plot presenting the prognostic risks associated with matrix‐producing CAF (matCAF) biomarkers across various cancer types, all showing significant results (*p* < .05). (C and D) Two matCAF cell lines, specifically expressing COL10A1, were successfully identified from gastric cancer patient tissues. In comparison to normal fibroblast (NF) cell lines, the matCAF cell lines exhibit elevated COL10A1 expression at both the RNA (C) and protein (D) levels. (E) A schematic representation of a three‐dimensional co‐culture system comprising NF or matCAF cell lines and organoid derived from gastric cancer patients. This co‐culture system was bifurcated into two groups: one group co‐cultured with the NF cell lines, and the other with the matCAF cell lines. (F) The left panel portrays the microscopic observations after a span of 15 days in co‐culture, indicating that matCAF promotes the growth of patient‐derived organoids within the co‐culture system, in contrast to the NF cell lines. The right panel demonstrates that in organoids co‐cultured with matCAFs, there is a reduction in the expression of p‐pRb and CDK6 proteins and an upregulation of p27 protein expression. (G) Co‐expression of the progenitor CAF (proCAF) marker OGN and myofibroblastic CAF (myCAF) marker ACTA2 in the same STAD cases. Cases #1–3 demonstrate high proCAF and relatively low myCAF expression. Conversely, cases #4–6 show a predominance of myCAFs with scarce detection of proCAF, indicating the heterogeneity of CAFs. High expression of both OGN and ACTA2 in CAFs implies a poor prognosis for STAD patients (scale bar = 200 μm for low magnification and 50 μm for high magnification), corroborating the survival data from The Cancer Genome Atlas (TCGA) dataset.

Our research then turned to address a crucial question: Do these CAF classifications hold clinical relevance? To investigate this, we gathered clinical data and gene expression levels from cancer patients in TCGA cohort. Patients were categorised into groups based on high or low expression of biomarkers for each CAF subtype, and survival analyses were performed. Results showed that while biomarkers for iCAF, myCAF and proCAF produced variable or inconclusive outcomes regarding their prognostic value, high expression of matCAF biomarkers consistently correlated with poorer survival across all cancer types examined (Figures 6A and S11). This highlights the potential of matCAF biomarkers as indicators of prognosis and as targets for therapy (Figure 6B).

Furthermore, we identified two matCAF cell lines from gastric cancer patient tissues, specifically expressing COL10A1 (Figure 6C,D). Compared to NF cell lines, these matCAF cell lines showed higher COL10A1 expression at both RNA and protein levels. We developed a 3D co‐culture system using either NF or matCAF cell lines with organoids derived from gastric cancer patients (Figure 6E). After 15 days of co‐culture, microscopic analysis indicated that matCAFs significantly enhanced the growth of patient‐derived organoids, unlike NF cell lines (Figure 6F, left panel). Additionally, organoids co‐cultured with matCAFs exhibited reduced expression of p‐pRb and CDK6 proteins and increased p27 protein expression (Figure 6F, right panel).

In the context of STAD, nearly all CAF subtype biomarkers showed a negative correlation with patient survival, suggesting their role in promoting gastric carcinogenesis. This was further validated by conducting OGN and ACTA2 IHC staining on STAD tissue microarrays. We observed varying predominance of either proCAF or myCAF in different cases. This heterogeneity among CAFs emphasises the potential of enrichment of either proCAF or myCAF as a marker of poor prognosis in STAD patients (Figure 6G and Table S1). These insights could offer a fresh viewpoint on the biological behaviour of these CAF subtypes and contribute to create more efficient treatment approaches in the future.

### 3.8 Screening and validation of drugs targeting matCAF

**FIGURE 7:**
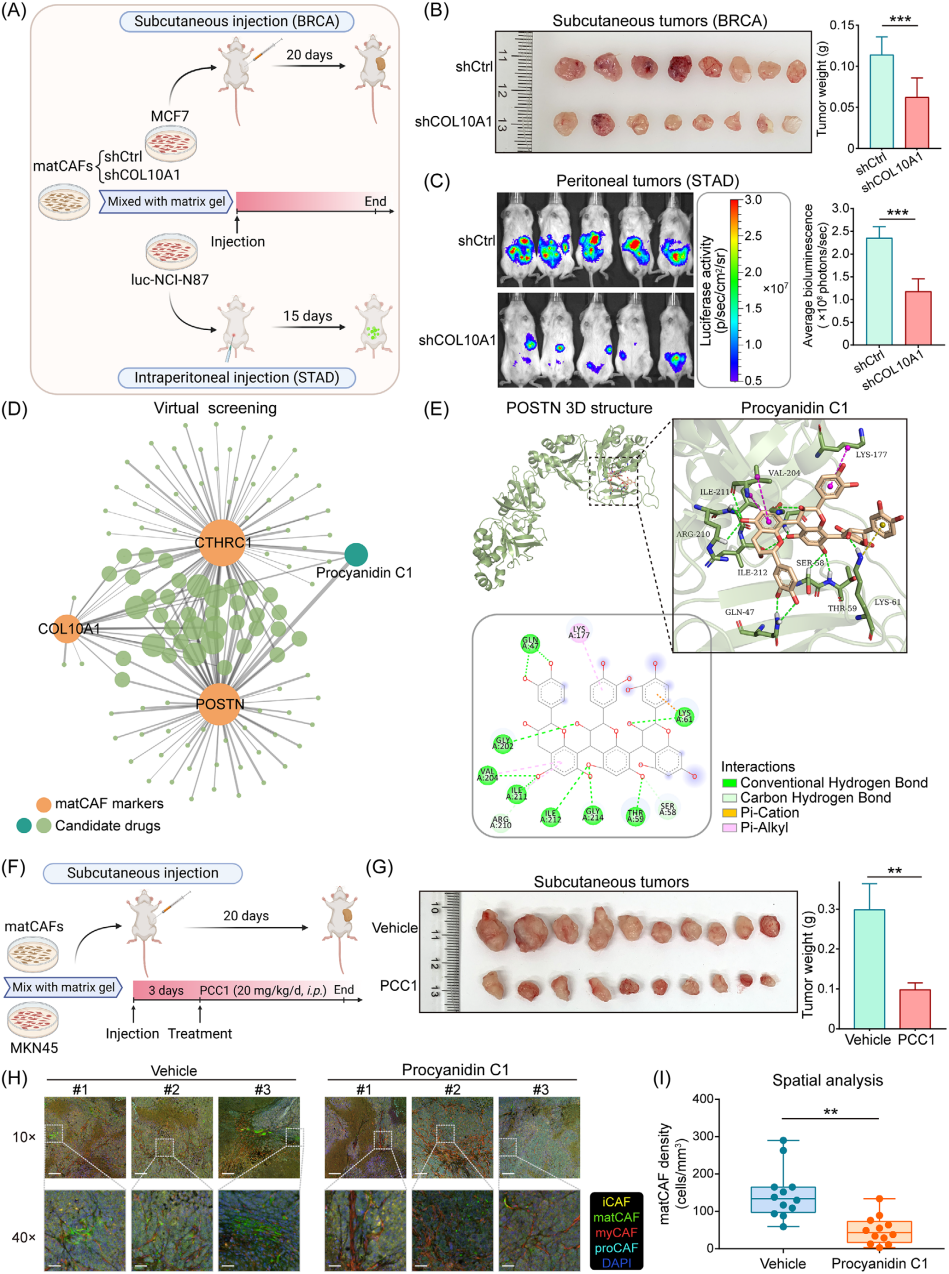
Inhibition of matrix‐producing cancer‐associated fibroblast (matCAF) suppresses tumour growth and metastasis. (A) Schematic representation of the in vivo mouse model, which involved co‐injecting matCAF and tumour cells. The upper segment depicts the BRCA mouse model with a subcutaneous injection of MCF7 cells, while the lower segment shows the STAD mouse model with an intraperitoneal injection of luc‐NCI‐N87 cells. In both experiments, the matCAF were divided into two groups: the shCtrl group and the shCOL10A1 group, depending on the treatment received. (B) Images of subcutaneous tumours illustrate that tumour growth in the BRCA model was notably inhibited in the shCOL10A1 group (*n* = 8/group). Histogram statistics reveal that the tumour weight in the shCOL10A1 group was significantly smaller than that in the shCtrl group (*p* < .001). (C) In vivo imaging and histogram analysis of bioluminescence levels in mice demonstrate that COL10A1 knockdown in matCAF diminished the capacity for STAD abdominal metastasis (*n* = 5/group; *p* < .001). (D) Procyanidin C1 (PCC1) was predicted to be effective against all three matCAF markers. (E) The predicted three‐dimensional structure and intermolecular interactions of POSTN binding to PCC1 are shown. (F) Visual observation of mice after subcutaneous inoculation with CAFs and MKN45 cells, which were combined with matrix gel. (G) Images of subcutaneous tumours in the vector and PCC1 groups. Tumour weight in the PCC1 group was significantly lower than in the control group (*n* = 10/group; *p* < .01). (H) The multiplex immunofluorescence (mIF) images of different CAF subtype biomarkers in the vector and PCC1 groups. Green staining was reduced in the PCC1 group compared with the vector group (scale bar = 200 μm for low magnification and 50 μm for high magnification). Yellow represents CCL2 (inflammatory CAF [iCAF] biomarker); green represents COL10A1 (matCAF biomarker); red represents ACTA2 (myofibroblastic CAF [myCAF] biomarker); cyan represents OGN (progenitor CAF [proCAF] biomarker); blue DAPI represents nuclei. (I) Four random fields of view in each stained section were selected, and matCAF density was calculated via spatial analysis. The analysis revealed that matCAF cell density in the PCC1 treatment group was significantly lower than in the vehicle group (*p* < .01).

In light of the cancer‐promoting role of matCAF, we performed in vivo experiments using a mouse model. This involved co‐injecting matCAF and tumour cells (Figure 7A). In the BRCA model, tumour proliferation was largely impeded in the group with COL10A1 knockdown matCAFs, as evidenced by notably smaller tumour weights compared with control group (Figure 7B). In the gastric cancer (STAD) model, in vivo imaging and bioluminescence analysis showed that matCAF with COL10A1 knockdown substantially reduced STAD's abdominal metastasis capacity (Figure 7C).

Further, we reviewed and compiled current drugs with the potential to target CAF biomarkers (Table S9). This table outlines several CAF biomarkers as potential candidates for drug targeting, with associated medications either in existence or undergoing clinical trials. This indicates the possibility of targeting CAFs to mitigate malignancy in solid tumours. Yet, it is important to note that these drugs primarily focus on singular biomarkers. Due to the poor prognosis associated with matCAF in patients with solid tumours, our objective was to identify a drug that could simultaneously target all three matCAF markers, enhancing matCAF eradication efforts. Virtual screening predicted PCC1 as a compound with strong binding affinity to all three matCAF marker proteins (Figure 7D and Table S10). Our drug‐target network, with orange nodes representing the three matCAF biomarkers (*CTHRC1*, *COL10A1* and *POSTN*) and green nodes indicating candidate chemicals, visualised these potential interactions. POSTN emerged as a viable candidate for drug development, given the feasibility of its drug‐target binding mechanism. Substantial intermolecular interactions were detected between PCC1 and 12 POSTN residues (Figure 7E). PCC1, known as a potent anti‐cancer compound and potential ageing retardant,^27^, ^54^ was not previously reported to inhibit matCAF.

To assess the pharmacodynamics of PCC1, we created a subcutaneous tumour mouse model using CAFs and MKN45 cells combined with matrix gel, which were then injected subcutaneously into NSG mice. In this model, tumours in the mice treated with PCC1 were markedly smaller tumours in comparison to controls (Figure 7F,G). Further, staining of tumour tissue sections after PCC1 treatment showed a significant reduction in matCAF density (Figure 7H,I). These results demonstrate that PCC1 inhibits matCAF and correlates with reduced tumour growth. Overall, our findings not only highlight the therapeutic potential of PCC1 but also emphasise the importance of targeting matCAF in cancer treatment strategies to suppress tumour progression.

## 4 DISCUSSION

The primary challenges in CAF research involve accurately characterising CAF heterogeneity and subpopulations, identifying distinct markers, as well as understanding the unique roles of each group. It is important to note that molecular classifications of CAFs vary across diverse types of cancer as suggested by various researchers.^43^, ^55^, ^56^, ^57^ Traditionally, CAF subpopulations have been categorised mainly into iCAFs and myCAFs, based on their predominant functions in different cancers.^46^, ^58^ This categorisation is exemplified by the induction of IL‐6^+^ CAFs (iCAF) and α‐SMA^+^ CAFs (myCAF) from quiescent pancreatic stellate cells in pancreatic ductal adenocarcinoma through IL‐1 and tumour growth factor‐beta (TGF‐β) stimulation.^59^, ^60^ In breast cancer research, one study classified CAFs into two populations: FAPP^+^/PPDPNP^+^P and FAPP^+^P/PPDPNP^−^ populations, with the former showing enrichment of TGF‐β signalling and a correlation with cancer advancement.^13^, ^61^ Kieffer et al. provided an extensive molecular classification of CAFs in breast cancer based on scRNA‐seq, identifying eight distinct CAF clusters, which included three iCAF and five myCAF types.^62^ Additionally, other research in melanoma, head and neck squamous cell carcinoma, and lung cancer further subdivided CAFs into six subtypes.^63^ Despite extensive research, a universally accepted molecular classification of CAFs is still lacking. Our study, utilising scRNA‐seq, has established four molecular CAF subtypes across a pan‐cancer spectrum: proCAF, iCAF, myCAF and matCAF. This classification is relevant to a variety of solid tumours and corresponds well with the predominant characteristics of each tumour type. Notably, we observed an almost complete absence of proCAF and matCAF in both LIHC and PRAD. While this could be due to limited sample sizes, it also suggests that the pronounced fibrosis observed during the progression of these cancer types may require further investigation.^39^, ^64^ Indeed, as a biomarker for myCAF, α‐SMA is one of the hallmark markers of tissue fibrosis. In the context of liver cancer, fibrosis is predominantly driven by myofibroblast cells. This phenomenon underpins the reason why myCAFs constitute a major proportion of the CAF subtypes in hepatic tumours. The presence of α‐SMA in these cells not only signifies their fibrogenic activity but also highlights their critical role in the TME.

In our single‐cell analysis, we discovered that matCAF are prominent in various solid tumours. matCAF's primary role is the secretion of large amounts of collagen, positioning it as a key organiser of the ECM. This is supported by the enrichment of specific highly expressed genes and distinct TF signatures. Collagen, a vital component of the cancer tissue matrix, has been recognised for its role in promoting tumour metastasis and progression, aligning with our observations.^65^, ^66^ PCA clustering analysis identified matCAF as a relatively conserved and stable subtype across most tumour types. Clinically, each CAF subtype's unique biomarker signatures indicate that high matCAF signatures correlate with poor prognosis in almost all adenocarcinomas. This finding leads to the exploration of strategies targeting matCAF. Notably, the ablation of COL10A1 in matCAF within cancer tissue significantly reduced tumour progression. Our computational virtual screening identified PCC1 as a potential targeting agent for three matCAF biomarkers: COL10A1, POSTN and CTHRC1. Using PCC1 in a subcutaneous tumour mouse model of gastric cancer showed marked inhibition of tumour growth and a significant decrease in matCAF. This aligns with previous research showing PCC1's role in slowing cellular senescence^27^ and impeding colorectal cancer progression.^67^

While some studies highlight the potential for CAF targeting to inadvertently accelerate cancer progression,^68^, ^69^ this underscores the diverse roles of various CAF subgroups within the TME. Our focus on matCAF, particularly its link with poorer prognoses in cancer patients due to its oncogenic properties, offers a nuanced perspective. A key function of matCAF is collagen secretion, enhancing ECM formation. Collagen I homotrimers, crucial ECM components, have been implicated as oncogenes in pancreatic cancer, actively contributing to tumour progression.^65^ However, it is important to recognise that in some contexts, specific CAFs might act as tumour suppressors through their ECM production. This highlights the complexity and heterogeneity in CAF biology, varying according to the CAF subtype and tumour context. Future research will delve deeper into the intrinsic oncogenic mechanisms of matCAF.

In our study, we observed that tumours linked with unfavourable survival and limited chemotherapy efficacy, referred to as ‘cold tumours’ such as LIHC and BRCA,^70^ had fewer iCAFs and more myCAFs. Conversely, ‘hot tumours’ characterised by increased inflammatory cell infiltration, such as STAD and COAD, displayed a higher presence of iCAFs, a subpopulation known to respond favourably to immunotherapy. Emerging research supports the idea that transitioning from ‘cold’ TME to immunogenic ‘hot’ TMEs can enhance the efficacy of immunotherapy, leading to improved treatment outcomes.^71^, ^72^ Our study contributes a novel perspective on assessing immunotherapy sensitivity in solid tumours by analysing the proportion of iCAFs. Interestingly, in ‘hot tumours,’ we found that iCAFs predominantly interact with immune cells through the CXCL12–CXCR4 axis. However, the role of iCAFs in tumours seems to be bidirectional. Some studies indicate their tumour‐promoting effects,^43^ while others suggest tumour‐suppressive roles.^73^ This highlights the need for further functional tests to clarify the role of the iCAF population and the importance of a more detailed subclassification to understand the complexities of CAF roles.

We also examined the evolutionary trajectory of CAF subtypes, uncovering a temporal pattern in their emergence within the tumour environment. proCAFs appeared as one of the earliest forms, followed by an increase in iCAFs. matCAFs and myCAFs emerged in the middle‐to‐late stages of tumour evolution. Analysis of multi‐cancer pathology sections revealed an increased frequency of matCAF and particularly myCAF in mid‐to‐late‐stage tumours, suggesting a dynamic shift in CAF subpopulations as tumours progress. This indicates that CAF subtypes are not static during tumourigenesis but evolve over time. Research has shown that senescent CAFs exhibit a senescence‐associated secretory phenotype (SASP), rich in pro‐inflammatory cytokines, and iCAFs have demonstrated robust performance under hypoxic conditions.^74^, ^75^ Given that senescence and hypoxia are prevalent throughout tumour progression, this provides a basis for the evolution of iCAFs. However, iCAFs in advanced‐stage tumours gradually diminish, corresponding with a reduced immune response. The shift towards matCAF and myCAF in later stages suggests a potential link between these subtypes and resistance to radiotherapy and chemotherapy in advanced tumours.^76^ While our study does not directly confirm this hypothesis, it aligns with previous findings and points to a new direction for future research. Understanding the specific roles of these CAF subtypes could be pivotal in developing targeted therapies, especially for advanced, treatment‐resistant tumours.

## 5 CONCLUSIONS

In conclusion, this study provides a more comprehensive understanding of the role of different CAF populations in tumour development. Our findings open up the potential to develop specific anti‐CAF therapeutic strategies targeting cancer‐promoting CAFs, based on the molecular features and functional roles of each CAF population.

## AUTHOR CONTRIBUTIONS

Wei Kang and Ka Fai To provided direction and guidance on the whole project. Bonan Chen and Wai Nok Chan drafted the manuscript. Bonan Chen, Wai Nok Chan, Fuda Xie, Chun Wai Mui, Xiaoli Liu, Alvin H.K. Cheung, Zhenhua Zhang, Canbin Fang and Peiyao Yu analysed the data. Raymond W.M. Lung, Chit Chow, Shihua Shi, Shikun Zhou, Guoming Chen, Zhangding Wang, Shouyu Wang, Xiaofan Ding, Bing Huang and Li Liang analysed the primary sample and provided bioinformatics support. Yujuan Dong, Chi Chun Wong, William K.K. Wu, Alfred S.L. Cheng, Nathalie Wong, Jun Yu, Kwok Wai Lo and Gary M.K. Tse reviewed the manuscript and made significant revisions. The final manuscript has been approved by all authors.

## CONFLICT OF INTEREST STATEMENT

The authors declare they have no conflicts of interest.

## ETHICS STATEMENT

All methods used in our studies involving human participants adhered strictly to the ethical guidelines of our institutional research committee, aligning with the principles outlined in the 1964 Helsinki declaration and its subsequent modifications, or equivalent ethical norms. Furthermore, all procedures involving animal handling and experimentation were conducted with the sanction of the Hong Kong Department of Health and under the ethical approval of the Animal Experimentation Ethics Committee at the Chinese University of Hong Kong.

## Supporting information

Supporting Information

Supporting Information

## Data Availability

The single‐cell expression data from our study have been made available in the NCBI Gene Expression Omnibus database under the respective accession codes: for BRCA—GSE161529; for CHOL—GSE138709 and GSE142784; for COAD—GSE132465; for LIHC—GSE112271; for LUAD—GSE131907; for NEPC—GSE137829; for PRAD—GSE141445; for OV and UCEC—GSE173682; and for UVM—GSE139829. The raw expression data for PAAD is stored in the Genome Sequence Archive database, available under accession code CRA001160. The raw expression data for STAD is provided by Stanford Medicine and can be accessed at https://doi.org/10.1158/1078‐0432.CCR‐19‐3231. Additionally, both the raw and processed data are downloadable from our dedicated website (www.cafatlas.com). For those interested in the specific methodologies, detailed codes used for scRNA analysis and figure generation are accessible at https://github.com/caf‐atlas/Rcodes.
