# Supplementary material for: The molecular classification of cancer‐associated fibroblasts on a pan‐cancer single‐cell transcriptional atlas
Source: Clin Transl Med. 2023 Dec 26;13(12):e1516. doi: 10.1002/ctm2.1516 (PMC10751516; doi:10.1002/ctm2.1516)
Supplement: Supplementary file 1 — Supporting Information [file CTM2-13-e1516-s002.docx]

**Supplemental information**

**The molecular classification of cancer-associated fibroblasts on a pan-cancer single-cell transcriptional atlas**

Bonan Chen^1,2,3,*^, Wai Nok Chan ^1,2,3,*^, Fuda Xie^1,2,3^, Chun Wai Mui^1,2,3^, Xiaoli Liu^1,2,3^, Alvin H.K. Cheung^1^, Raymond W.M. Lung^1^, Chit Chow^1^, Zhenhua Zhang^4^, Canbin Fang^1^, Peiyao Yu^5^, Shihua Shi^6^, Shikun Zhou^7^, Guoming Chen^8^, Zhangding Wang^9^, Shouyu Wang^10^, Xiaofan Ding^11^, Bing Huang^12^, Li Liang^5^, Yujuan Dong^13^, Chi Chun Wong^2^, William K.K. Wu^14^, Alfred S.L. Cheng^15^, Nathalie Wong^13^, Jun Yu^2,16^, Kwok Wai Lo^1^, Gary M.K. Tse^1^, Wei Kang^1,2,3,§^, and Ka Fai To^1,2,3,§^


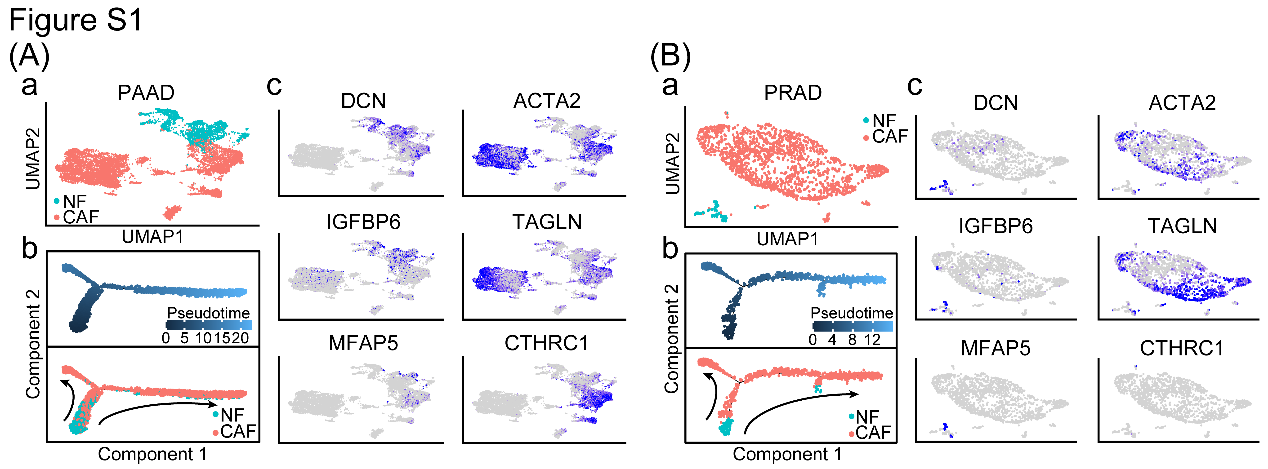


**Figure S1. Discrimination between NF and CAF within the fibroblast population, related to Figure 1.** (A) a, A UMAP plot illustrating the distinction between NF and CAF in pancreatic cancer. b, Monocle2 was employed to infer the developmental trajectories of NF and CAF in BRCA. The upper panel shows a pseudotime plot, while the lower panel displays the NF and CAF classifications. The pseudotime analysis suggests a developmental progression from NF to CAF. c, Expression levels of NF biomarkers (*DCN*, *IGFBP6*, and *MFAP5*) and CAF biomarkers (*ACTA2*, *TAGLN*, and *CTHRC1*) exhibit distinct distributions in respective NF and CAF clusters. (B) Similar plots are shown for prostate cancer.

**
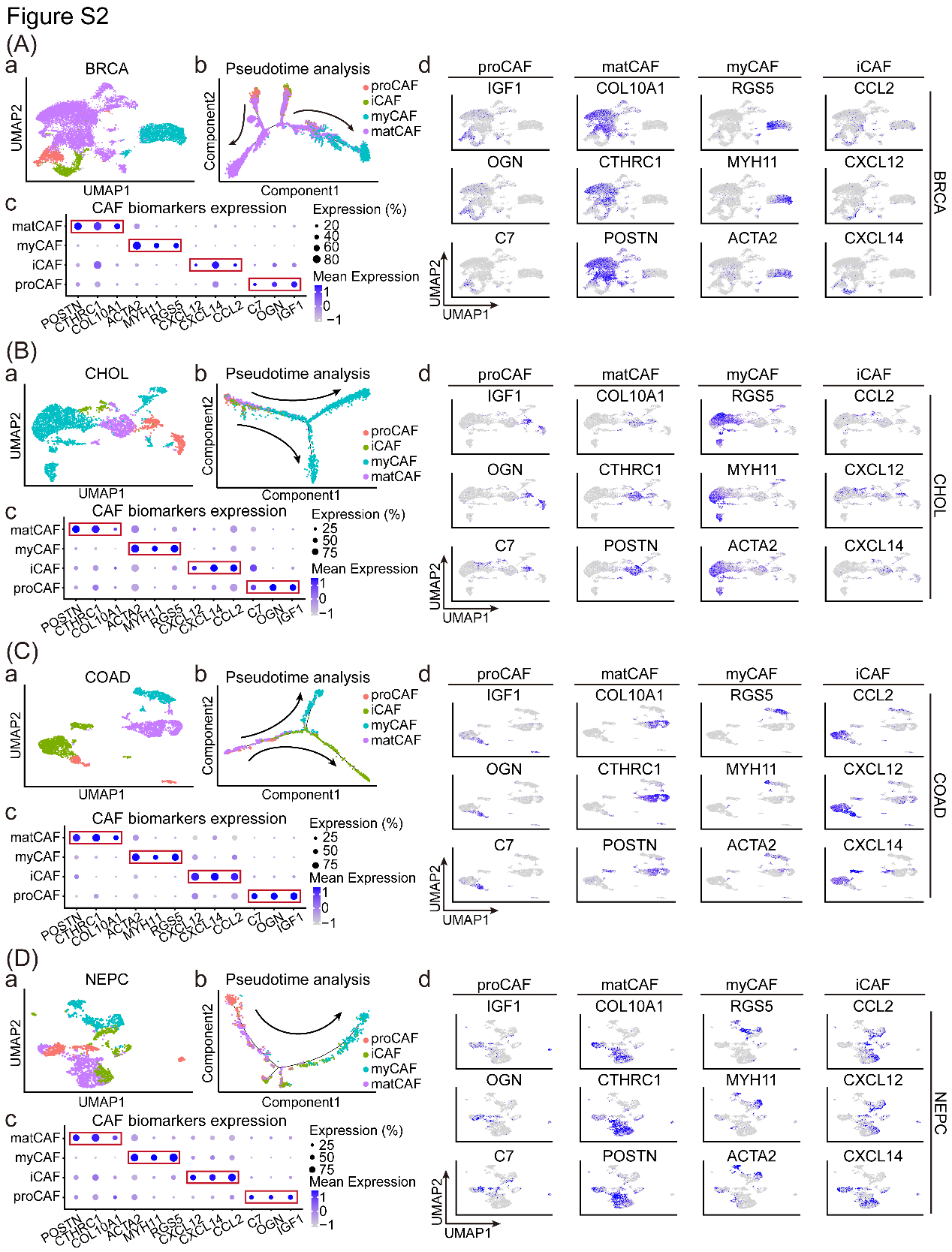
**

**Figure S2. Classification of four CAF subtypes and their specific biomarkers, related to Figure 2.** (A) a, UMAP plot demonstrating the classification and distribution of proCAF, iCAF, myCAF, and matCAF in BRCA. b, A developmental trajectory of CAF subtypes in BRCA was identified, indicating that proCAF differentiates into other CAF subtypes. c, Within their respective CAF subtype groups, specific biomarkers exhibit a higher proportion and elevated average expression levels. d, UMAP plots of biomarker expression levels of different CAF subtypes in BRCA, including proCAF (*IGF1*, *OGN*, and *C7*), matCAF (*COL10A1*, *CTHRC1*, and *POSTN*), myCAF (*RGS5*, *MYH11*, and *ACTA2*) and iCAF (*CCL2*, *CXCL12*, and *CXCL14*). Notably, biomarkers with higher expression levels align with their respective CAF subtype populations. (B) Similar plots are presented for CHOL. (C) Corresponding plots are given for COAD. (D) Comparable illustrations are provided for NEPC.

**
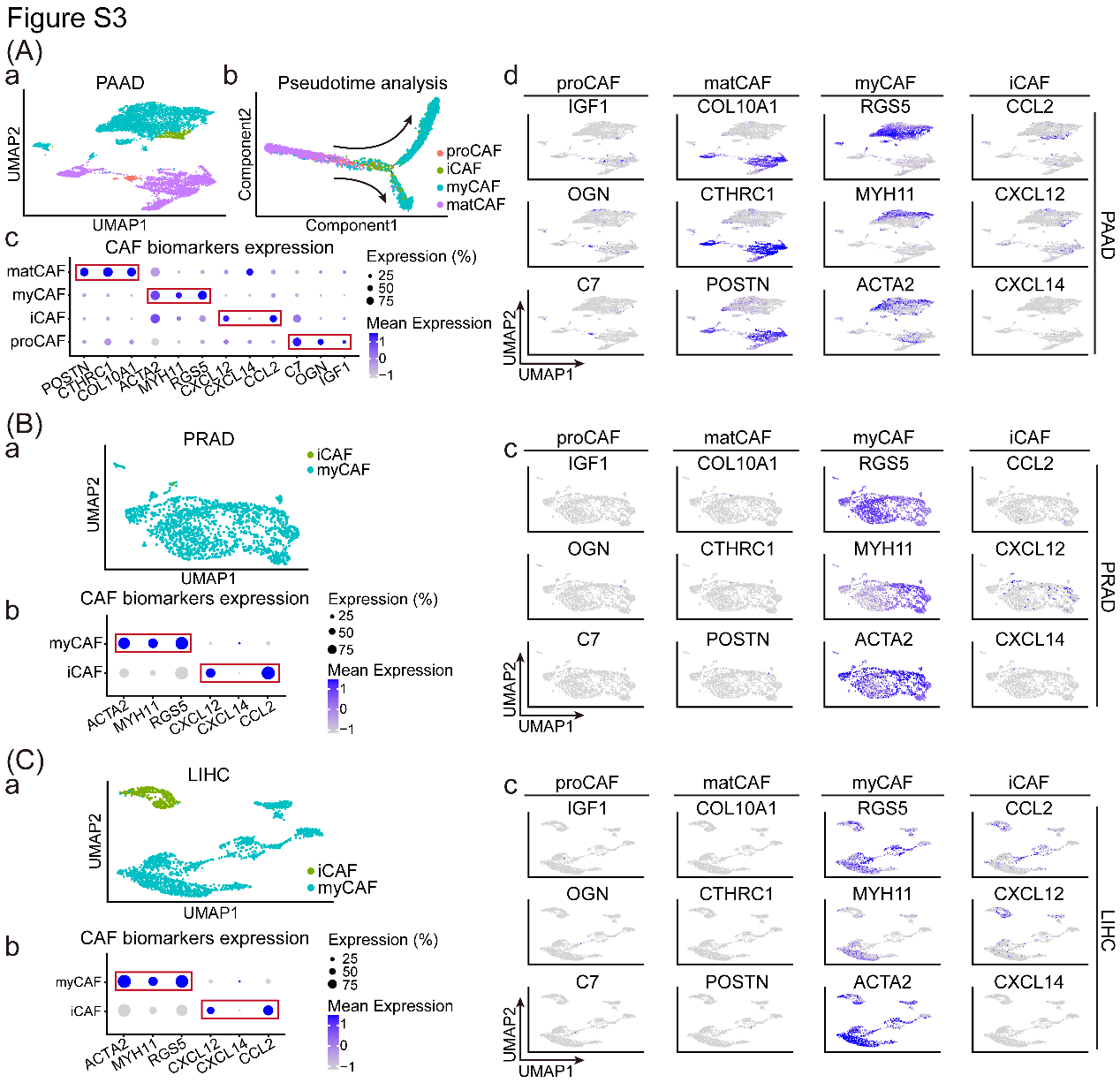
**

**Figure S3. Classification and identification of four CAF subtypes and their specific biomarkers (continued), in relation to Figure 2.** (A) a, UMAP plot presenting the categorization and distribution of proCAF, iCAF, myCAF, and matCAF in PAAD. b, The developmental progression of CAF subtypes in PAAD is portrayed, suggesting that proCAF evolves into the other CAF subtypes. c, Distinct biomarkers display higher proportions and elevated average expression levels in their respective CAF subtype groups. d, UMAP plots reflect the expression levels of biomarkers for different CAF subtypes in PAAD. It’s noteworthy that the biomarkers’ distributions essentially align with their corresponding CAF subtype populations. (B) A corresponding plot for PRAD. (C) A similar plot for LIHC. Importantly, the single-cell datasets for PRAD and LIHC did not exhibit presence of proCAF and matCAF subtypes.

**
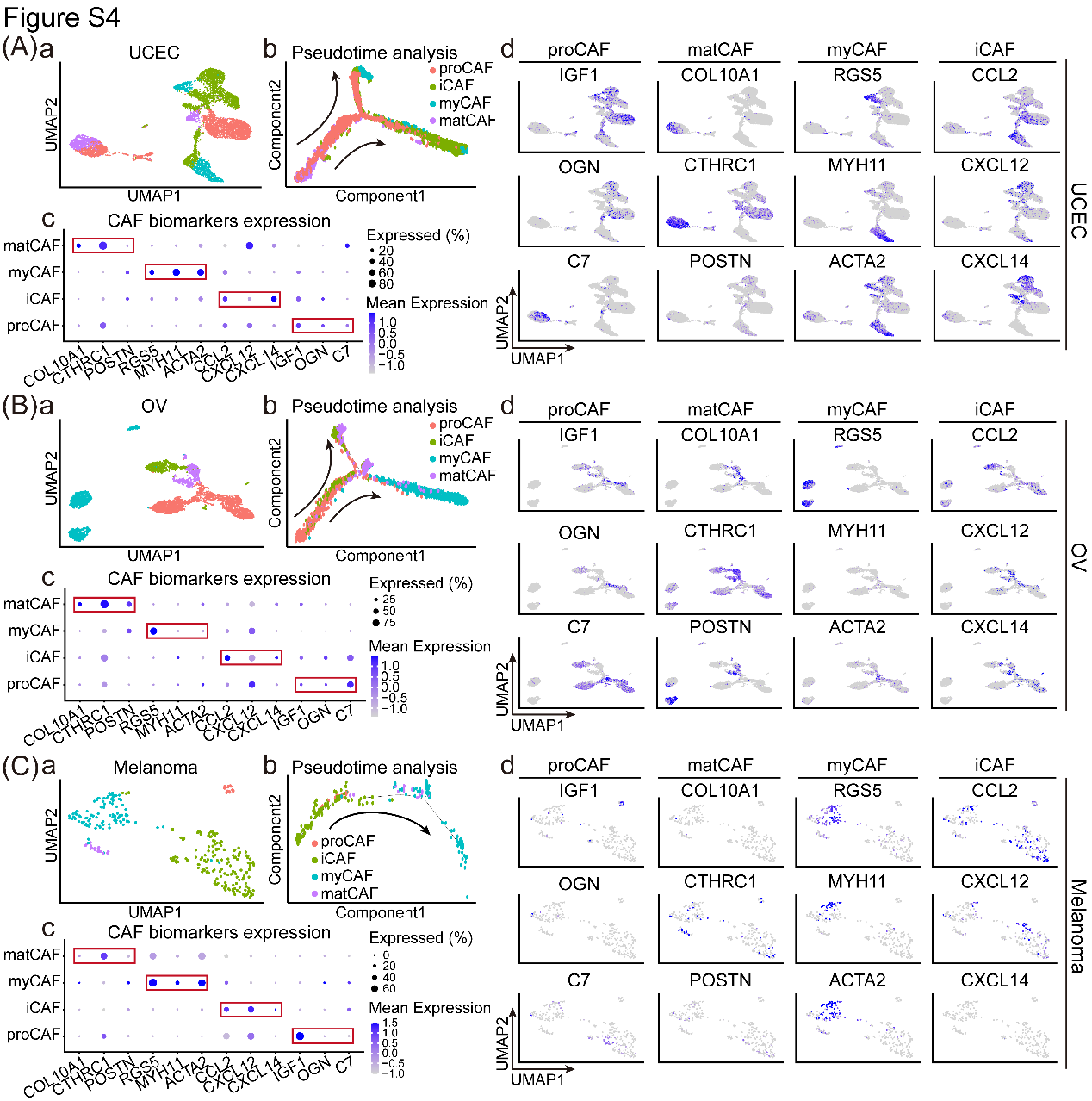
**

**Figure S4. Classification and identification of four CAF subtypes and their specific biomarkers (continued), in relation to Figure 2.** (A) A series of plots analogous to those in Figure S2A, depicting UCEC. These include a UMAP plot illustrating the categorization and distribution of proCAF, iCAF, myCAF, and matCAF in UCEC, along with a demonstration of the developmental trajectory of CAF subtypes and a display of specific biomarkers with higher proportions and elevated mean expression levels in the respective CAF subtype groups. (B) A corresponding series of plots for OV. (C) A parallel set of plots for Melanoma.

**
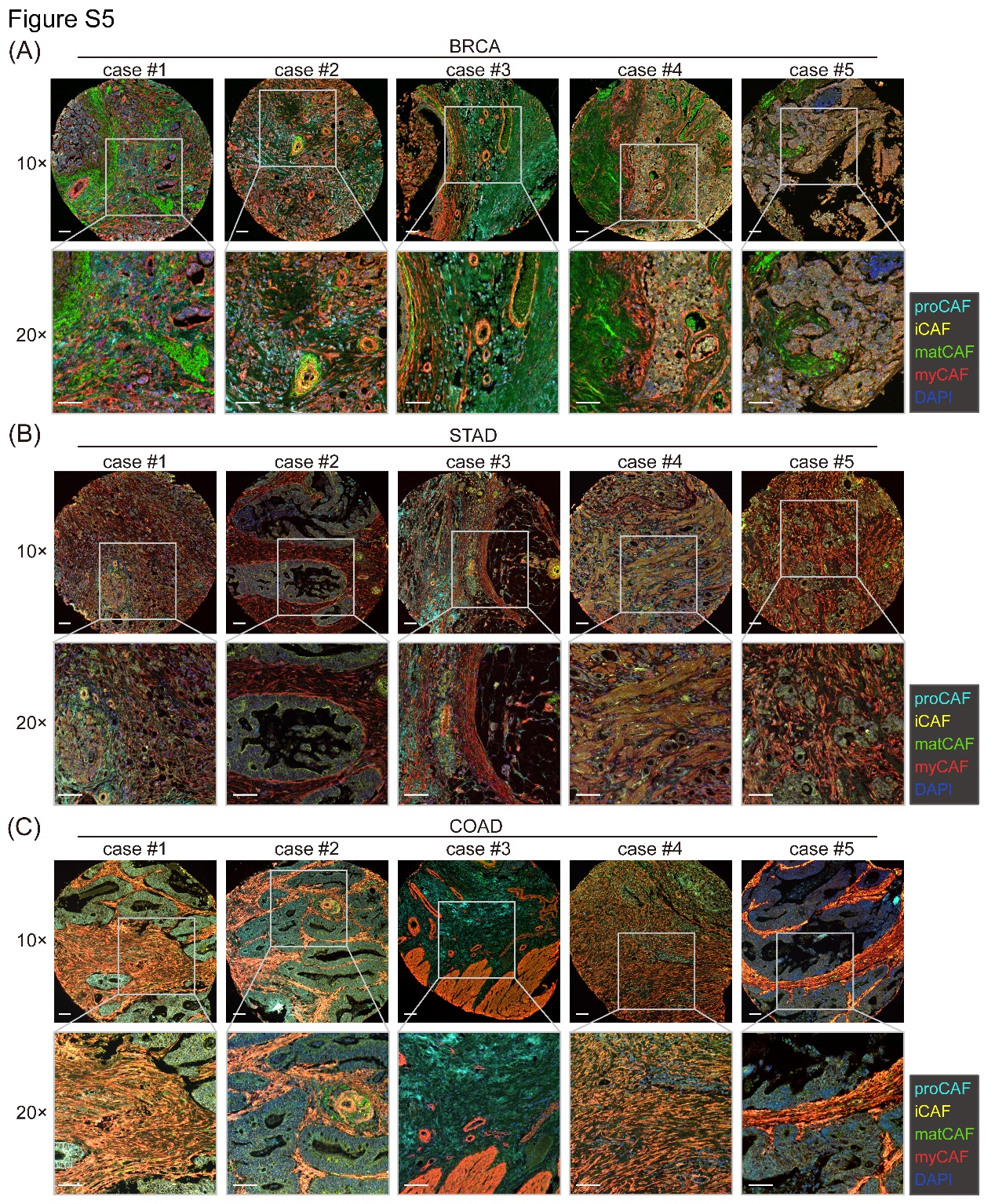
**

**Figure S5. Independent coexistence of distinct CAF subtypes in the tumor microenvironment, related to Figure 2.** (A) The mIHC staining displaying COL10A1 (green), CCL2 (yellow), OGN (cyan), and α-SMA (red), along with DAPI staining (blue) in BRCA tissue samples, representative of n=5 patients. For each of the five cases, the upper panel showcases images captured at a 10x magnification, while the lower panel presents images from the same tissue captured at a 20× magnification. The area of interest in the 10× image is demarcated by a gray dashed line. The scale bar in all images represents 200 μm. (B, C) Exhibiting representative images of identical mIHC staining across a spectrum of STAD and COAD samples extracted from a tissue microarray. Consistently, all scale bars in these images represent 200 μm.

**
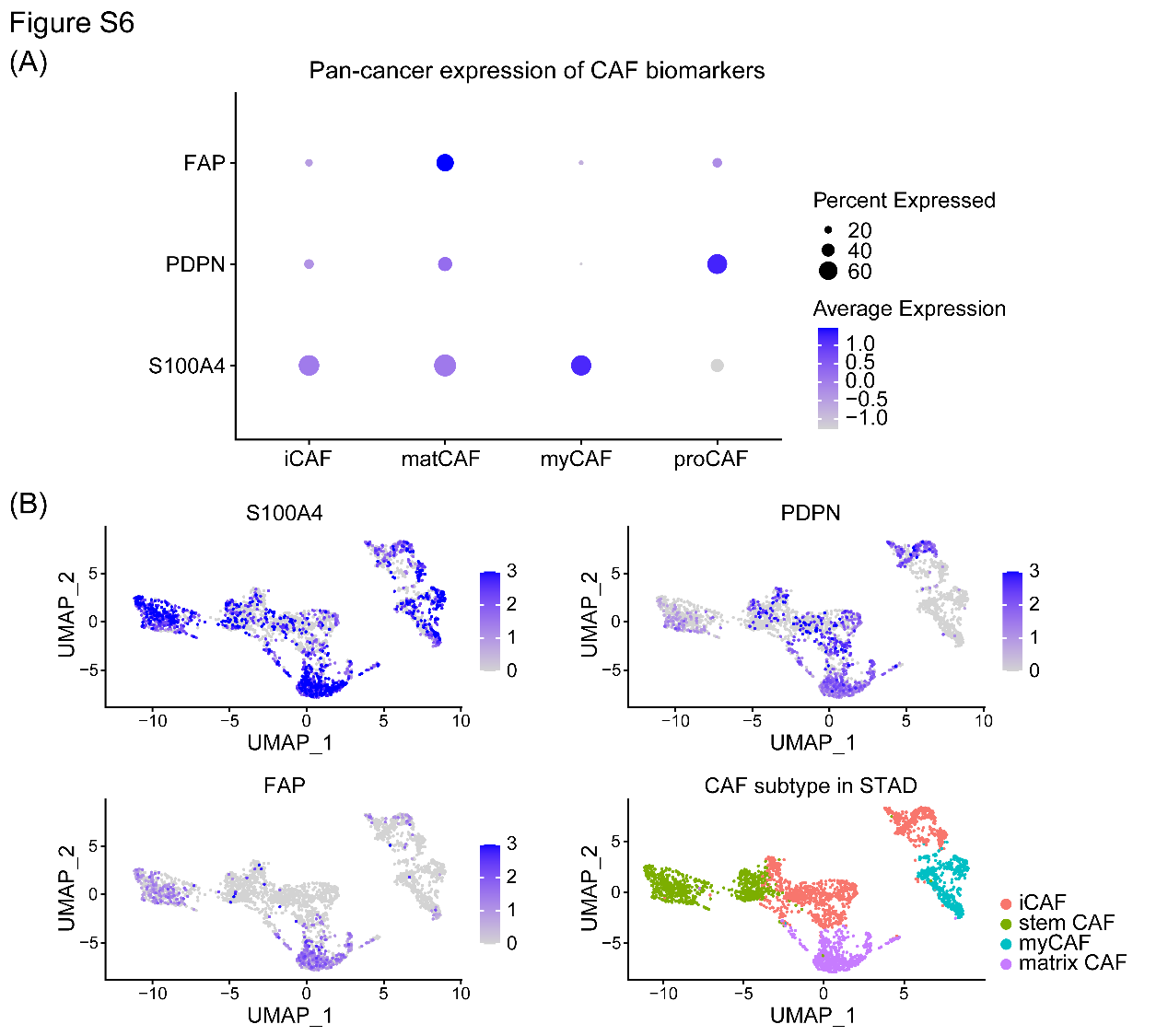
**

**Figure S6. Expression patterns of the other CAF biomarkers across pan-cancer single-cell analyses.** (A) Dot plot representation of RNA expression levels for CAF biomarkers, including FAP, PDPN, and S100A4 among different cancer types. (B) Feature plot demonstrates the diverse expression profiles of FAP, PDPN, and S100A4 across various CAF subtypes in STAD.

**
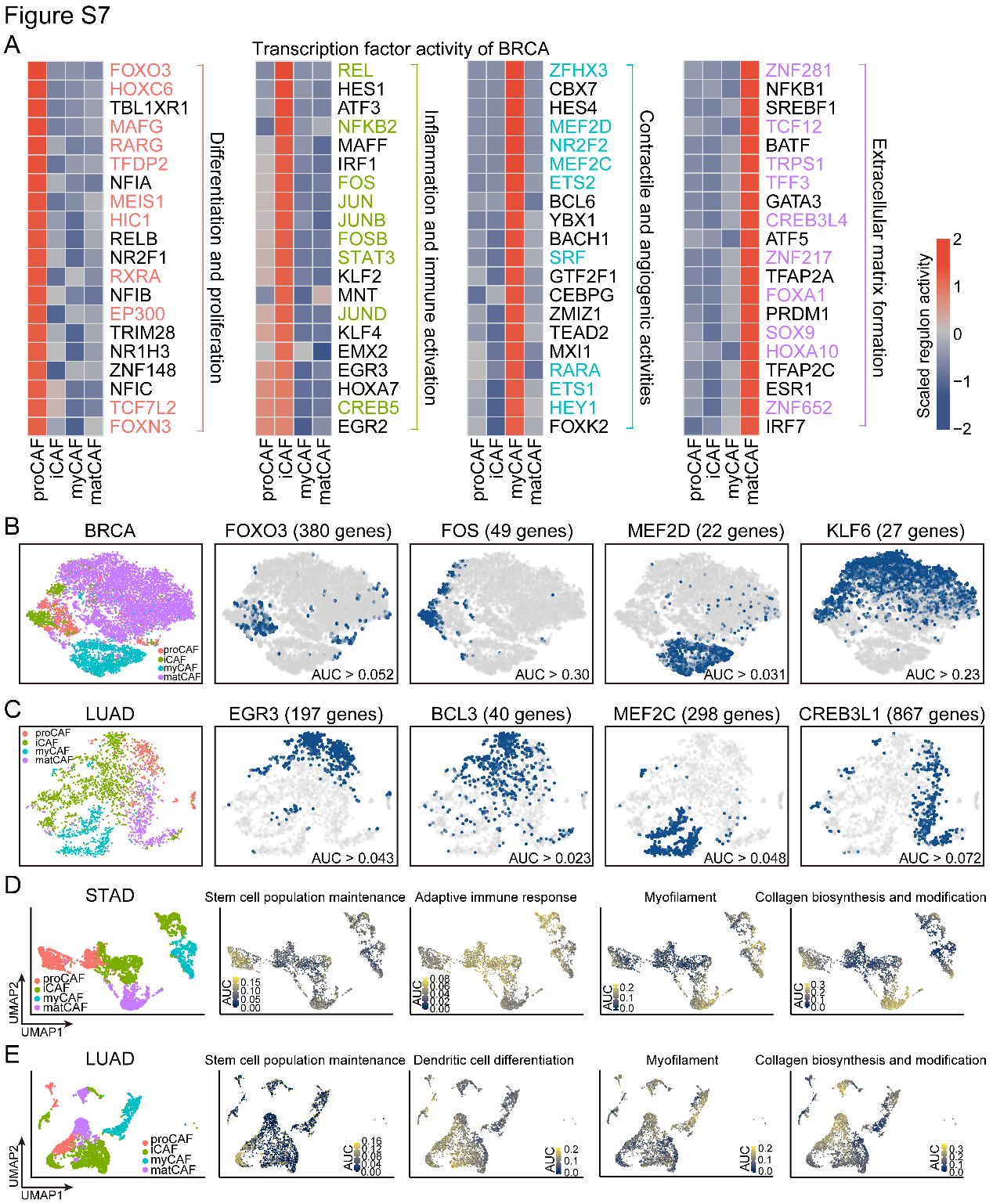
**

**Figure S7. Multiple functional roles of CAF subtypes within single-cell distribution, related to the Figure 3.** (A) The activation levels of the top 20 transcription factors for each CAF subtype in BRCA are depicted. Transcription factors with roles corresponding to the CAF subtypes are emphasized. (B) The leftmost t-SNE plot illustrates the distribution of each CAF subtype. Subsequent four t-SNE plots, generated through SCENIC analysis based on the binary activity matrix of specific transcription factors, are displayed in dark blue for each CAF subtype, elucidating distinct CAF functions in BRCA. (C) SCENIC analysis was also executed for CAFs in LUAD, yielding consistent results.

**
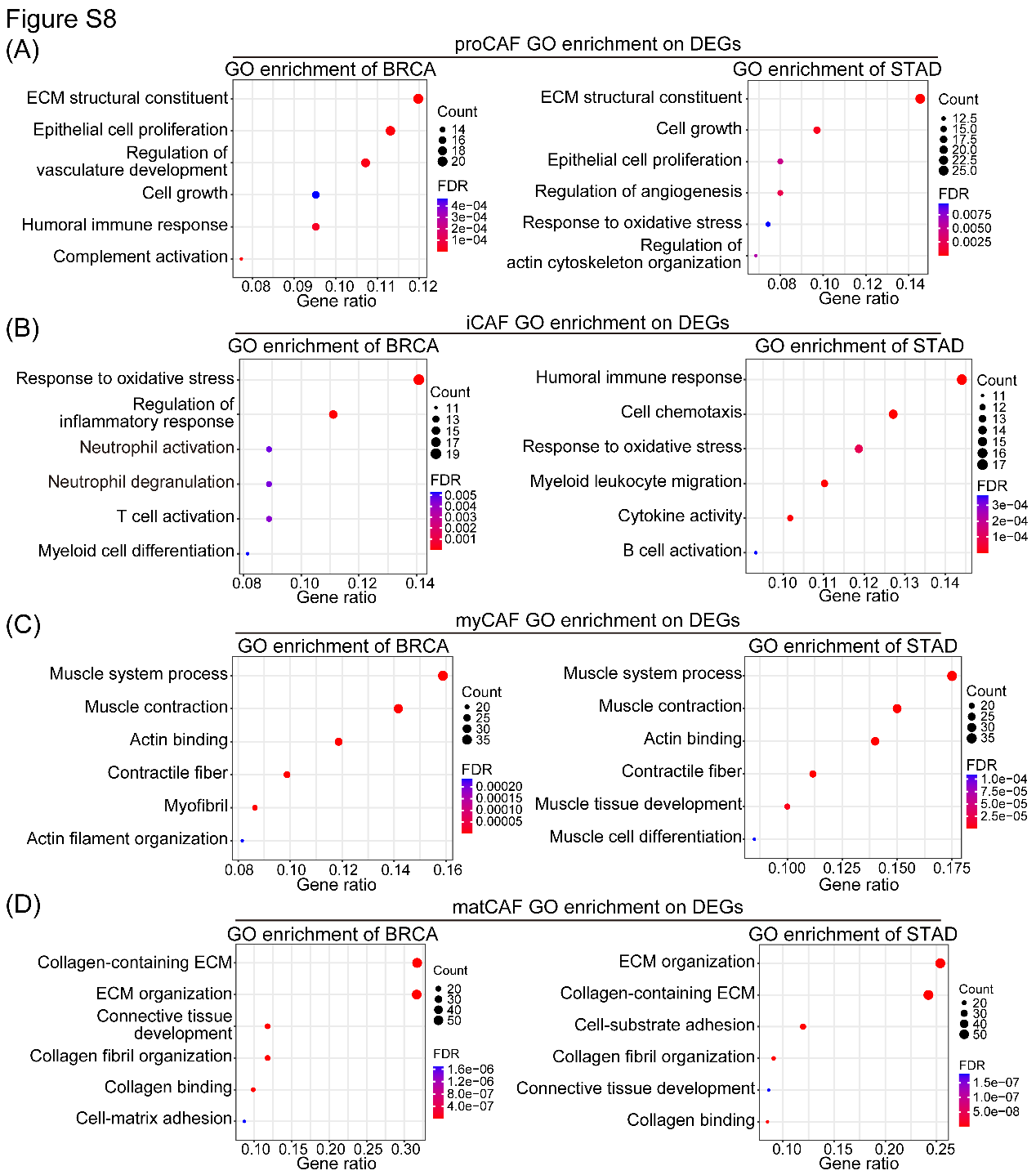
**

**Figure S8. GO enrichment pathways across different CAF subtypes in BRCA and STAD, related to Figure 3.** (A) The DEG analysis was conducted between proCAF and other CAF subtypes, with genes featuring a log_2_|fold change| > 1.5 and *P* < 0.05 selected for GO enrichment analysis. Results from both BRCA and STAD were congruent, indicating that proCAF was involved in proliferation-related pathways. (B) The same GO enrichment analysis, maintaining the same fold change and *P*-value criteria, was performed for the remaining CAF subtypes in BRCA and STAD. Pathways related to immune responses were notably enriched in iCAF. (C) Pathways associated with contractile and muscle-related activities were markedly enriched in myCAF. (D) Genes involved in ECM formation were predominantly enriched in matCAF. (E) The functional roles of CAF subtypes were validated using AUCell analysis in STAD. Four gene sets, associated respectively with stemness, immune responses, myofilament, and ECM formation, were chosen for AUCell analysis on CAFs in STAD. Each cell received an AUC score indicative of the relative expression of the signature genes and the strength of related activities. Cells with high AUC scores aligned with the corresponding CAF subtype population indicated in the leftmost UMAP plot. (F) AUCell analysis using identical gene sets was carried out with CAFs in LUAD, yielding results in harmony with STAD observations.

**
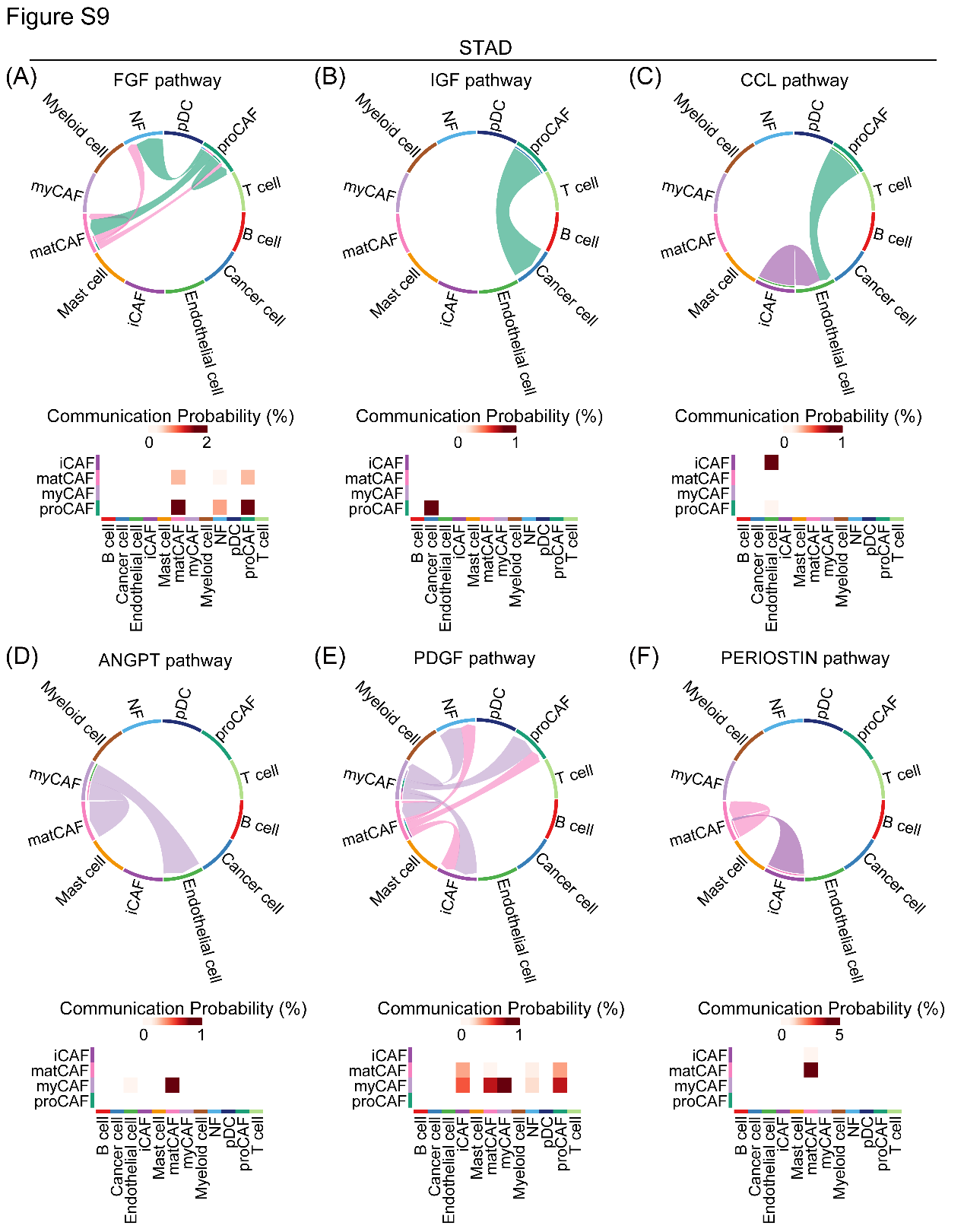
**

**Figure S9. Involvement of CAF subtypes in intercellular communication networks within STAD, related to Figure 5.** (A) The chord diagrams depict the intercellular communication networks from the CAF scRNA-seq dataset of STAD. Accompanying heatmaps provide a visual representation of the communication probability, a measure of relative signaling strength, of each cell-cell interaction. The FGF pathway principally serves as a communication channel among proCAF, NF, and matCAF cells. (B) The IGF signaling pathway is predominantly derived from proCAF and ultimately received by cancer cells. (C) Immune signaling via the CCL pathway primarily originates from iCAF and proCAF. (D) The ANGPT pathway, on the other hand, is mainly sourced from myCAF. (E) In a similar fashion, the PDGF pathway represents a dominant communication network between myCAF and matCAF with other cells. (F) The PERIOSTIN pathway, existing in matCAF, exhibits an autocrine form of communication.

**
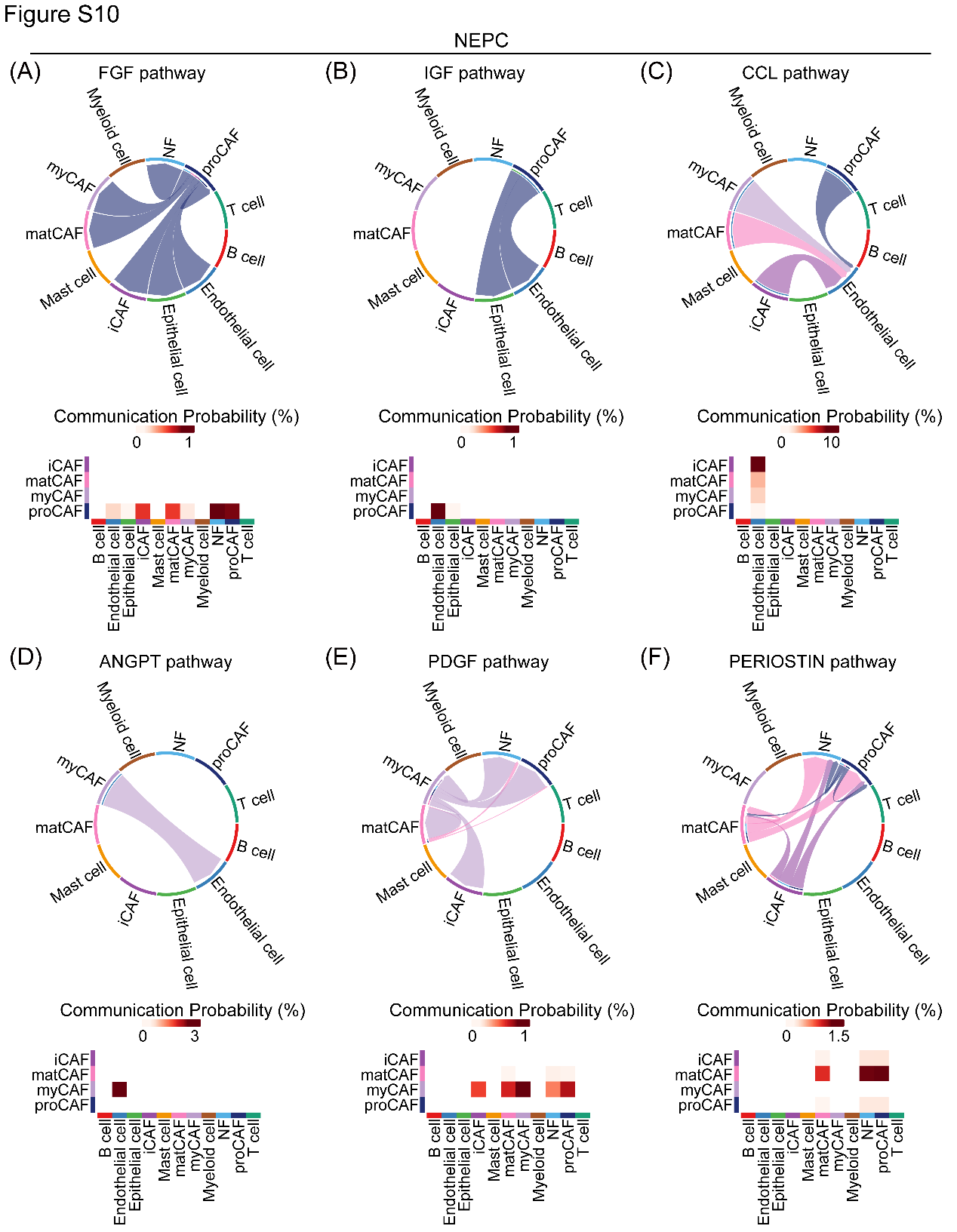
**

**Figure S10. Participation of CAF subtypes in intercellular communication networks within NEPC, associated with Figure 5.** (A) Chord diagrams visualize the intercellular communication networks derived from the CAF scRNA-seq dataset of NEPC. Accompanying heatmaps serve to depict the communication probability, a measure of relative signaling strength, of each cell-cell interaction. Predominantly, the FGF pathway serves as a communication conduit among proCAF, NF, and matCAF cells. (B) The IGF signaling pathway mainly originates from proCAF and is ultimately targeted to cancer cells. (C) Immune signaling via the CCL pathway primarily originates from iCAF and proCAF. (D) The ANGPT pathway is primarily sourced from myCAF. (E) Similarly, the PDGF pathway constitutes a significant communication network between myCAF and matCAF with other cells. (F) The PERIOSTIN pathway, present within matCAF, represents an autocrine mode of communication.

**
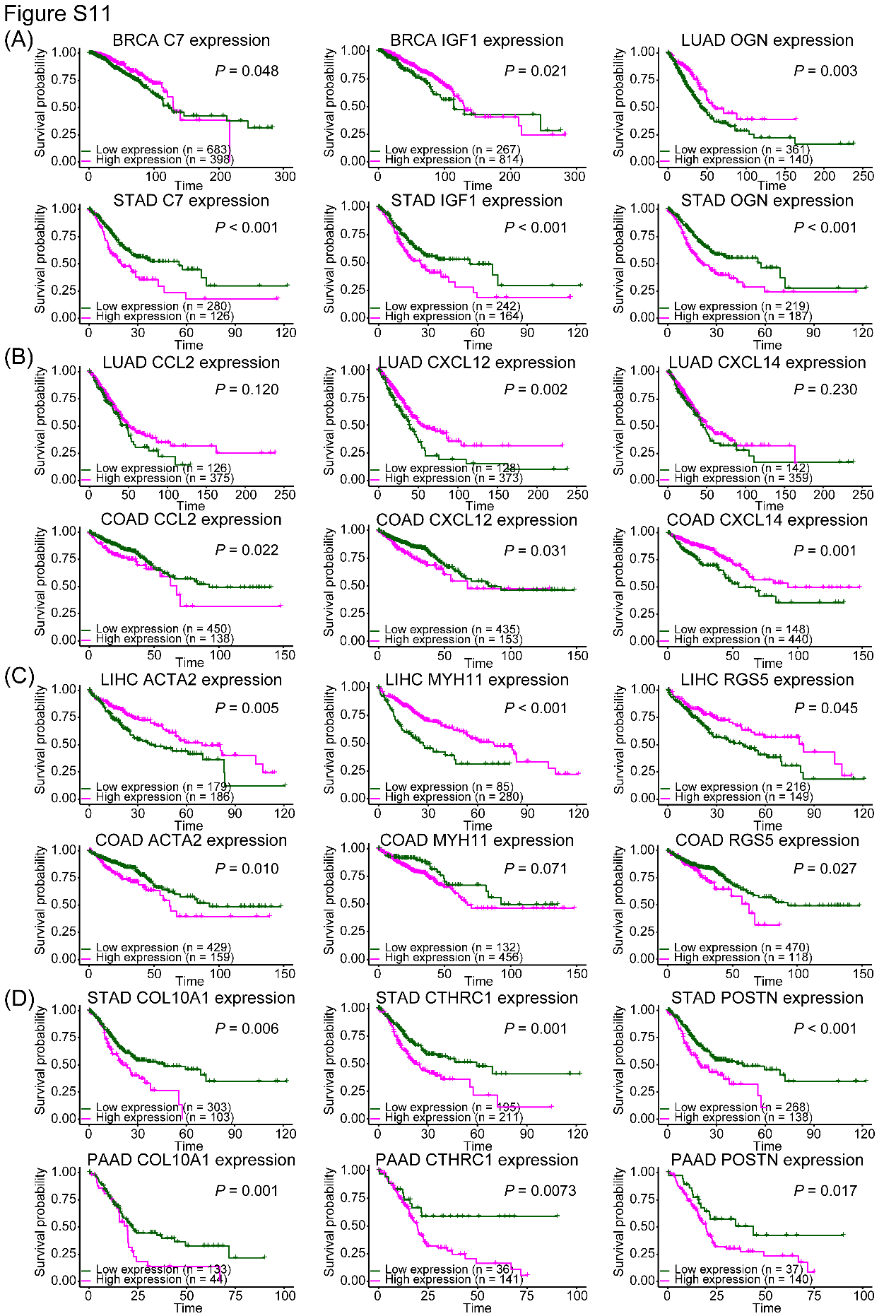
**

**Figure S11. Varied implications of CAF subtypes in clinical prognosis, corresponding to Figure 6.** Patients from various cancer types in the TCGA cohort were segregated into two groups based on the optimal cut-off point: one group exhibiting higher expression levels of CAF biomarkers and the other demonstrating lower expression levels. Survival analysis between these two groups unveiled that the clinical prognosis linked with biomarkers of CAF subtypes was not consistent across cancer types. Representative survival curves display the prognostic significance of biomarkers associated with proCAF (A), iCAF (B), myCAF (C), and matCAF (D), respectively.

**Table S1. Patient information corresponding to representative images of STAD tissue microarray**

| **Case No.** | **Related to** | **Gender** | **Age** | **Grade** | **Stage** | **TNM stage** | ***H.pylori*** |
| --- | --- | --- | --- | --- | --- | --- | --- |
| #1 | Figure 2C | Male | 72 | 2 | IV | T4N2M0 | Positive |
| #1 | Figure 4H | Female | 74 | 1 | Ib | T2N0M0 | Positive |
| #2 | Figure 4H | Male | 68 | 1 | II | T2N1M0 | Positive |
| #3 | Figure 4H | Female | 58 | 2 | IIIa | T2N2M0 | Positive |
| #4 | Figure 4H | Male | 49 | 3 | IV | T3N3M1 | Positive |
| #1 | Figure 6G | Male | 72 | 3 | IIIa | T3N1M0 | Negative |
| #2 | Figure 6G | Female | 73 | 2 | IV | T1N3M0 | Positive |
| #3 | Figure 6G | Male | 64 | 3 | IV | T4N3M1 | Negative |
| #4 | Figure 6G | Male | 42 | 3 | II | T2N1M0 | Positive |
| #5 | Figure 6G | Male | 65 | 2 | II | T2N1M0 | Positive |
| #6 | Figure 6G | Female | 58 | 2 | IIIa | T2N2M0 | Positive |
| #1 | Figure S5B | Male | 53 | 3 | IV | T3N1M0 | Negative |
| #2 | Figure S5B | Male | 72 | 2 | IIIa | T3N3M0 | Positive |
| #3 | Figure S5B | Male | 75 | 2 | IIIb | T3N2M0 | Positive |
| #4 | Figure S5B | Male | 68 | 2 | II | T2N1M0 | Positive |
| #5 | Figure S5B | Male | 64 | 2 | II | T2N1M1 | Positive |

**Table S2. Patient information corresponding to representative images of LUAD tissue microarray**

| **Case No.** | **Related to** | **Gender** | **Age** | **Stage** | **TNM stage** | **Smoke** |
| --- | --- | --- | --- | --- | --- | --- |
| #1 | Figure 2G | Female | 74 | IIIA | T3N1M0 | Yes |
| #1 | Figure 2M | Male | 47 | IVA | T3N0M1 | No |
| #2 | Figure 2M | Female | 81 | IIB | T3N0M0 | Yes |
| #3 | Figure 2M | Female | 65 | IVA | T2N0M1 | No |
| #4 | Figure 2M | Female | 76 | IIIA | T3N1M0 | Yes |
| #5 | Figure 2M | Male | 74 | IB | T2N0M0 | No |

**Table S3. Patient information corresponding to representative images of BRCA tissue microarray**

| **Case No.** | **Related to** | **Gender** | **Age** | **Grade** | **Stage** | **Diagnosis** |
| --- | --- | --- | --- | --- | --- | --- |
| #1 | Figure S5A | Female | 49 | 2 | IIIC | Invasive breast cancer |
| #2 | Figure S5A | Female | 46 | 2 | IIB | Invasive lobular carcinoma |
| #3 | Figure S5A | Female | 51 | 2 | IA | Invasive breast cancer |
| #4 | Figure S5A | Female | 51 | 3 | IIIC | Invasive breast cancer |
| #5 | Figure S5A | Female | 66 | 3 | IIB | Invasive breast cancer |

**Table S4. Patient information corresponding to representative images of COAD tissue microarray**

| **Case No.** | **Related to** | **Gender** | **Age** | **Grade** | **Stage** | **TNM stage** |
| --- | --- | --- | --- | --- | --- | --- |
| #1 | Figure 4H | Male | 63 | 2 | I | T2N0M0 |
| #2 | Figure 4H | Female | 53 | 2 | II | T3N0M0 |
| #3 | Figure 4H | Male | 66 | 2 | III | T4N1M0 |
| #4 | Figure 4H | Female | 57 | 2 | IV | T24N2M1 |
| #1 | Fgirue S5C | Male | 67 | 2 | IV | T4N2M1 |
| #2 | Figure S5C | Female | 74 | 2 | IV | T3N0M0 |
| #3 | Fgirue S5C | Female | 89 | 1 | III | T3N1M0 |
| #4 | Figure S5C | Male | 61 | 2 | II | T3N0M0 |
| #5 | Fgirue S5C | Male | 66 | 4 | IV | T4N2M1 |

**Table S9. CAF biomarkers as druggable targets**

| **Biomarker** | **Drug** | **Disease** | **Reference** |
| --- | --- | --- | --- |
| **proCAF** |  |  |  |
| IGF1 | Picropodophyllin | Osteosarcoma | ^1^ |
| OGN | Chrysophanol | Meningioma | ^2^ |
| C7 | Eculizumab* | Myasthenia gravis | ^3^ |
| **matCAF** |  |  |  |
| COL10A1 | Cyclopamine | Osteoarthritis | ^4^ |
| CTHRC1 | Cyclovirobuxine D | Colorectal cancer | ^5^ |
| POSTN | Anti-POSTN mAb | Breast cancer | ^6^ |
| **myCAF** |  |  |  |
| MYH11 | Blebbistatin | - | ^7^ |
| ACTA2 | Ferulic acid | Hepatocellular carcinoma | ^8^ |
| **iCAF** |  |  |  |
| CCL2 | 15a | Atherosclerosis | ^9^ |
| CXCL12 | Plerixafor | GI solid tumors | ^10^ |

* via blocking its upstream C5

**Table S10. Parameters of docking procedures**

| Parameter | Value |
| --- | --- |
| Energy grid box | 40×40×40Å |
| Energy grid spacing | 0.375Å |
| number of individuals in population | 150 |
| maximum number of energy evaluations | 2.5×10^6^ |
| maximum number of generations | 2.7×10^4^ |
| rate of gene mutation | 0.02 |

**MATERIALS AND METHODS**

**Gene Ontology (GO) enrichment analysis**

Differentially expressed genes (DEGs) were pinpointed using the “FindMarkers” function within the R package “Seurat” (version 4.0.2). This identification was based on the Wilcoxon Rank Sum test, complemented by Bonferroni correction. DEGs meeting the criteria of an adjusted P-value < 0.05 and a fold change > 1.5 were earmarked for subsequent analyses. Gene Ontology (GO) enrichment analysis was executed using the “enrichGO” function in the “clusterProfiler” R package (version 3.18.1) ^11^. We selected appropriate GO terms with a False Discovery Rate (FDR) ≤ 0.05 and depicted them using the “ggplot2” R package.

**Gene Set Enrichment Analysis (GSEA)**

Gene sets for our study were selected and obtained from the MSigDB database (software.broadinstitute.org/gsea/msigdb/collections.jsp). We conducted the enrichment analysis using the R package “AUCell” (version 1.12.0) ^12^. The process began with invoking the “AUCell_buildRankings” function to prepare the dataset. Utilizing the gene sets downloaded as gene matrix transposed (GMT) files, we calculated the AUC score for each cell via the “AUCell_calcAUC” function. Subsequently, cells were color-coded and visualized in a UMAP plot using the ggplot2 package.

**Survival analysis**

Survival analysis in our study was conducted using the R package “survminer” (version 0.4.9). The Hazard Ratio (HR) was calculated through the Cox proportional hazards model, with a 95% Confidence Interval (CI) reported. The Kaplan-Meier survival curve was modeled using the survfit function. The “surv_cutpoint” function was employed to determine the optimal cutpoint for dividing a population into two groups based on their gene expression levels. This was achieved via the “maxstat.test” function of the R package “maxstat,” which identifies the maximum rank statistic through repeated iterations. We set the “minprop” parameter to 0.2 to guarantee that each group comprised at least 20 percent of the total population. Kaplan-Meier survival curves were then plotted using both “survfit” and “ggsurvplot” functions. The two-sided log-rank test was utilized for comparing Kaplan-Meier survival curves.

**qRT-PCR and Western blot analysis**

In our research, qRT-PCR was implemented to assess changes in mRNA expression levels. We used the QuantStudio 7 Flex Real-Time PCR System, in combination with TB Green Premix Ex Taq (#RR420A, TAKARA, Otsu, Japan), for this analysis. β-actin served as the reference gene for normalizing gene expression levels, and relative differences in gene expression were determined using the 2^-ΔΔCt^ method. The setup for the PCR reaction included: 5.0 μl of SYBR^®^ Premix Ex Taq, 0.2 μl of both forward and reverse primers, 2.0 μl of diluted cDNA template, and 2.6 μl of RNase-free DECP H2O, culminating in a total volume of 10.0 μl per reaction. The primers used were as follows: GAGGCTATCCAGCGTACTCCA (*B2M* forward), CGGCAGGCATACTCATCTTTT (*B2M* reverse), ATGCTGCCACAAATACCCTTT (*COL10A1* forward), GGTAGTGGGCCTTTTATGCCT (*COL10A1* reverse).

For Western blot assays, proteins were extracted from cells using RIPA lysis buffer during a 30-minute incubation on ice. The BCA Protein Assay Kit (#23225, Thermo Scientific, Waltham, MA, USA) was utilized to quantify the total protein concentration. Following protocol ^13^ from earlier publications, protein samples were separated via 10% or 15% SDS-PAGE and then transferred to PVDF membranes. These membranes were blocked for 2 hours using TST buffer (a mix of Tris-Buffered Saline and 0.1% Tween 20 Detergent) with 5% non-fat milk added. The membranes were then incubated overnight at 4°C with primary antibodies, followed by two rinses with TBST. This was succeeded by a further 1-hour incubation with appropriate HRP-linked secondary antibodies. Protein bands were visualized using enhanced chemiluminescence, allowing for the detection and analysis of protein expression related to our study's focus.

**Molecular docking**

In our study, molecular docking was employed to evaluate the binding affinity between anti-cancer agents and matCAF marker proteins, utilizing Autodock 4.2.6 ^14^. Our library consisted of 4511 anti-cancer small molecules, with their structural data sourced from PubChem ^15^ (https://pubchem.ncbi.nlm.nih.gov/). The 3D structural files for CTHRC1, COL10A1, and POSTN were acquired from AlphaFold ^16^ (https://alphafold.ebi.ac.uk/). Prediction of active sites in matCAF markers was conducted using PrankWeb ^17^ (https://prankweb.cz/) and DeepSite ^18^ (https://www.playmolecule.com/deepsite/). The molecular docking process incorporated the Lamarckian genetic algorithm (LGA), and the details of this method are elaborated in Table S10. The docking outcomes were assessed based on the computed binding energy (BE), with compounds displaying a BE less than -8.18 kcal/mol (equivalent to an inhibition constant of 1 μM) identified as potential binders to the target proteins. We then constructed and visualized the drug-target network using Cytoscape 3.9.1 ^19^. The 3D structure of POSTN and binding site analysis of procyanidin C1 (PCC1, CAS: 37064-30-5) were visualized by PyMOL 2.3.

**References**

1 Wu, Q. *et al.* IGF1 receptor inhibition amplifies the effects of cancer drugs by autophagy and immune-dependent mechanisms. *J Immunother Cancer* **9**, doi:10.1136/jitc-2021-002722 (2021).

2 Wang, J. & Lv, P. Chrysophanol inhibits the osteoglycin/mTOR and activats NF2 signaling pathways to reduce viability and proliferation of malignant meningioma cells. *Bioengineered* **12**, 755-762, doi:10.1080/21655979.2021.1885864 (2021).

3 Zelek, W. M. & Morgan, B. P. Monoclonal Antibodies Capable of Inhibiting Complement Downstream of C5 in Multiple Species. *Front Immunol* **11**, 612402, doi:10.3389/fimmu.2020.612402 (2020).

4 Salem, O. *et al.* Naproxen affects osteogenesis of human mesenchymal stem cells via regulation of Indian hedgehog signaling molecules. *Arthritis Res Ther* **16**, R152, doi:10.1186/ar4614 (2014).

5 Jiang, F. *et al.* Cyclovirobuxine D inhibits colorectal cancer tumorigenesis via the CTHRC1AKT/ERKSnail signaling pathway. *Int J Oncol* **57**, 183-196, doi:10.3892/ijo.2020.5038 (2020).

6 Field, S. *et al.* Novel highly specific anti-periostin antibodies uncover the functional importance of the fascilin 1-1 domain and highlight preferential expression of periostin in aggressive breast cancer. *Int J Cancer* **138**, 1959-1970, doi:10.1002/ijc.29946 (2016).

7 Sirigu, S. *et al.* Highly selective inhibition of myosin motors provides the basis of potential therapeutic application. *Proc Natl Acad Sci U S A* **113**, E7448-E7455, doi:10.1073/pnas.1609342113 (2016).

8 Mu, M. *et al.* Ferulic acid attenuates liver fibrosis and hepatic stellate cell activation via inhibition of TGF-beta/Smad signaling pathway [Corrigendum]. *Drug Des Devel Ther* **13**, 1819, doi:10.2147/DDDT.S215949 (2019).

9 Bot, I. *et al.* A novel CCR2 antagonist inhibits atherogenesis in apoE deficient mice by achieving high receptor occupancy. *Sci Rep* **7**, 52, doi:10.1038/s41598-017-00104-z (2017).

10 Daniel, S. K., Seo, Y. D. & Pillarisetty, V. G. The CXCL12-CXCR4/CXCR7 axis as a mechanism of immune resistance in gastrointestinal malignancies. *Semin Cancer Biol* **65**, 176-188, doi:10.1016/j.semcancer.2019.12.007 (2020).

11 Ashburner, M. *et al.* Gene ontology: tool for the unification of biology. The Gene Ontology Consortium. *Nat Genet* **25**, 25-29, doi:10.1038/75556 (2000).

12 Aibar, S. *et al.* SCENIC: single-cell regulatory network inference and clustering. *Nat Methods* **14**, 1083-1086, doi:10.1038/nmeth.4463 (2017).

13 Chen, B. *et al.* Fangchinoline inhibits non-small cell lung cancer metastasis by reversing epithelial-mesenchymal transition and suppressing the cytosolic ROS-related Akt-mTOR signaling pathway. *Cancer Lett* **543**, 215783, doi:10.1016/j.canlet.2022.215783 (2022).

14 Morris, G. M. *et al.* AutoDock4 and AutoDockTools4: Automated docking with selective receptor flexibility. *Journal of computational chemistry* **30**, 2785-2791, doi:10.1002/jcc.21256 (2009).

15 Kim, S. *et al.* PubChem Substance and Compound databases. *Nucleic acids research* **44**, D1202-1213, doi:10.1093/nar/gkv951 (2016).

16 Varadi, M. *et al.* AlphaFold Protein Structure Database: massively expanding the structural coverage of protein-sequence space with high-accuracy models. *Nucleic acids research* **50**, D439-d444, doi:10.1093/nar/gkab1061 (2022).

17 Jendele, L., Krivak, R., Skoda, P., Novotny, M. & Hoksza, D. PrankWeb: a web server for ligand binding site prediction and visualization. *Nucleic acids research* **47**, W345-w349, doi:10.1093/nar/gkz424 (2019).

18 Jiménez, J., Doerr, S., Martínez-Rosell, G., Rose, A. S. & De Fabritiis, G. DeepSite: protein-binding site predictor using 3D-convolutional neural networks. *Bioinformatics (Oxford, England)* **33**, 3036-3042, doi:10.1093/bioinformatics/btx350 (2017).

19 Shannon, P. *et al.* Cytoscape: a software environment for integrated models of biomolecular interaction networks. *Genome research* **13**, 2498-2504, doi:10.1101/gr.1239303 (2003).
